# Temperature and intrinsic Ca^2+^ reshape TRPM4 pharmacology

**DOI:** 10.1038/s41594-026-01818-3

**Published:** 2026-06-09

**Authors:** Jinhong Hu, Sofia Ievleva, Sung Jin Park, Junuk Lee, Jie Cheng, Garrett O’Dea, Jiangnan Sheng, Juan Du, Wei Lü

**Affiliations:** 1 Department of Molecular Biosciences, Northwestern University, Evanston, IL USA; 2 Department of Pharmacology, Feinberg School of Medicine, Northwestern University, Chicago, IL USA; 3 Chemistry of Life Processes Institute, Northwestern University, Evanston, IL USA

**Keywords:** Cryoelectron microscopy, Transient receptor potential channels

## Abstract

Proteins operate in dynamic environments where ions, lipids and temperature collectively define their properties, yet most studies rely on simplified conditions that overlook these intrinsic variables. Here we show two such factors—temperature and Ca^2+^—remodel the function and pharmacology of TRPM4, an ion channel implicated in cardiac conduction, immune regulation, cancer and intestinal-fluid homeostasis. At physiological temperature and Ca^2+^, TPPO—previously considered a selective TRPM5 inhibitor inactive toward TRPM4—potently activates TRPM4, revealing strong synergy among temperature, Ca^2+^ and ligand binding. By contrast, Necrocide-1, a necroptotic activator targeting the same binding pocket, defies this logic: it opens TRPM4 without Ca^2+^ but is antagonized by Ca^2+^. Meanwhile, the inhibitors NBA and CBA engage a nearby pocket, locking the channel in a non-conductive pre-open state. Our findings highlight that even rigid binding pockets can exhibit temperature-dependent ligand recognition, revealing hidden pharmacology and informing selective, environment-aware therapeutic strategies.

## Abstract (web summary)

Hu et al. show that physiological temperature and intrinsic Ca^2+^ reprogram TRPM4 pharmacology, revealing hidden ligand activities missed under simplified assay conditions—even at structurally stable sites—and highlighting the need for environment-aware drug discovery.

## Main

Protein activity is inherently governed by the cellular environment in which it operates. A wide array of factors—including ions, lipids, temperature, pH and protein–protein interactions—collectively determine how a protein functions in its native context. Yet, because of technical constraints, most biophysical and pharmacological studies have been conducted under simplified conditions, which have yielded foundational insights but often fail to capture the full physiological complexity.

Increasing efforts now aim to bridge this gap by incorporating more native variables—for example, using nanodiscs, liposomes or membrane-derived vesicles to preserve native lipidic environments for membrane proteins^1–4^, and purifying proteins from native sources to retain endogenous protein compositions and modifications^5–8^. Despite these advances, one fundamental determinant, temperature, is largely overlooked in mechanistic and pharmacological studies, despite influencing every protein in the human body, in which proteins operate at 37 °C. Because most in vitro assays are performed at room temperature or lower, therefore, the broad impact of temperature on protein conformation, ligand recognition, efficacy and mechanism remains unexplored, with only a few recent exceptions^9,10^.

Alongside temperature, intracellular Ca^2+^ concentration is another intrinsic physiological variable that has pervasive influence over protein behavior. Although its role is not universal, Ca^2+^ often acts as a selective yet broad regulator of cellular physiology. It is among the most tightly controlled cellular cofactors, with cytosolic concentrations fluctuating dynamically from tens of nanomolar at rest to low micromolar during signaling, rising to abnormally high levels under pathological conditions such as ischemia, excitotoxicity or cancer^11^. Many proteins are directly regulated by Ca^2+^ through dedicated binding sites, and others respond indirectly through Ca^2+^-dependent signaling cascades. Although the structural and functional roles of Ca^2+^ have been extensively characterized, its impact on protein–ligand interactions—particularly in combination with other intrinsic factors, such as temperature—has rarely been considered. This is an important gap in our understanding of how Ca^2+^-regulated proteins engage ligands under physiological and pathological conditions.

The Ca^2+^-activated, temperature-sensitive channel transient receptor potential melastatin 4 (TRPM4) is an ideal model to investigate how intrinsic physiological factors influence drug responses^12–15^. TRPM4 is a non-selective monovalent cation channel whose activation by elevated cytosolic Ca^2+^ levels—arising from receptor-mediated signaling, Ca^2+^ entry through other channels or release from intracellular stores—leads to Na^+^ influx and membrane depolarization. This depolarization activates downstream depolarization-dependent mechanisms, including the regulation of voltage-gated ion channels, and thereby modulates diverse downstream processes, such as cardiac conduction, insulin secretion, neuronal excitability, immune regulation and intestinal-fluid homeostasis^16–25^. Pathogenic variants have been implicated in Brugada syndrome, a genetic heart-rhythm disorder, and other inherited conduction disorders, as well as diverse cancers^26–34^. In this context, TRPM4 antagonists could be therapeutically beneficial in cardiac diseases in which excessive TRPM4 activity contributes to pathological depolarization and conduction defects, whereas TRPM4 agonists might be relevant in certain cancers in which TRPM4 is upregulated and in which enhanced channel activity has been associated with necrotic cell-death pathways. Despite this broad physiological and pathological relevance, TRPM4 has remained largely untargeted by clinical pharmacology, with the single exception of bisacodyl—a widely used drug for chronic constipation—and its active metabolite deacetyl bisacodyl, which we recently identified and characterized as clinically relevant TRPM4 agonists^25^.

Our previous structural and functional studies have revealed that TRPM4 adopts two major conformations, a ‘cold’ and a ‘warm’ state, whose equilibrium is governed jointly by temperature and cytosolic Ca^2+^ concentration^10^. In the cold conformation, Ca^2+^ binds exclusively to an agonist site in the S1–S4 bundle (Ca_TMD_), giving rise to strongly outwardly rectifying currents. By contrast, in the warm conformation, the intracellular domain undergoes a pronounced rearrangement that is accompanied by Ca^2+^ binding at an additional intracellular site (Ca_warm_ or Ca_ICD_), resulting in currents that are less outwardly rectifying and exhibit substantial inward conductance. Together, these findings suggest that biophysical and pharmacological assays that are performed under room temperature and undefined Ca^2+^ conditions might not accurately capture the channel’s behavior under physiological settings.

Here, we systematically examine TRPM4 pharmacology under conditions that incorporate both temperature and Ca^2+^. Through combined cryogenic electron microscopy (cryo-EM) and electrophysiology, we reveal that these intrinsic factors profoundly shape ligand recognition, efficacy and mechanism. Our results identify the S1–S4 domain as a dynamic regulatory hub that integrates environmental and chemical cues to govern activation and inhibition. More broadly, this work establishes an environment-aware framework for pharmacology, in which incorporating intrinsic variables exposes hidden drug activities and new mechanisms of regulation. For widely expressed proteins such as TRPM4, these principles open opportunities to design therapeutics that exploit pathological local conditions—such as abnormally elevated Ca^2+^ levels or altered thermal states—for selective action in disease while preserving normal physiological function and minimizing side effects.

## Results

### Temperature reveals hidden pharmacology of TRPM4

During the cold-to-warm transition of TRPM4, Ca^2+^ and temperature drive major conformational changes in the intracellular domain, but only subtle shifts in the transmembrane domain^10^. From these structures, we identified three categories of ligand-binding sites that emerge exclusively in the warm conformation of the intracellular domain^10^. Although this outcome might be anticipated from the magnitude of intracellular domain (ICD) rearrangement, it nevertheless raises two central questions: whether drug screening performed exclusively at room temperature could have overlooked active compounds, and whether temperature-dependent ligand recognition is confined to regions undergoing large conformational changes (such as the intracellular domain) or extends to regions with more modest movements (such as the transmembrane domain).

To address these questions, we examined several previously reported TRPM4 ligands, as well as compounds deemed inactive against TRPM4. We focused on hydrophobic ligands predicted to interact with the transmembrane domain, including triphenylphosphine oxide (TPPO), necrocide-1 (NC1), and the anthranilic acid derivatives CBA (4-chloro-2-[2-(2-chloro-phenoxy)-acetylamino]-benzoic acid) and NBA (4-chloro-2-(2-(naphthalene-1-yloxy) acetamido) benzoic acid). TPPO, initially identified as a selective TRPM5 inhibitor with a half-maximal inhibitory concentration (IC_50_) of 12 μM, has shown no activity against TRPM4 in fluorescence-based membrane-potential assays^35^. NC1 has recently been characterized as a TRPM4 activator that promotes Na^+^ influx and triggers necrotic cell death^36^, suggesting it has potential therapeutic applications in cancer. NBA and CBA are currently the best-characterized TRPM4 inhibitors, with IC_50_ values in the sub-micromolar range^37^. Importantly, all prior functional and structural studies were conducted at room temperature or lower.

**Fig. 1:**
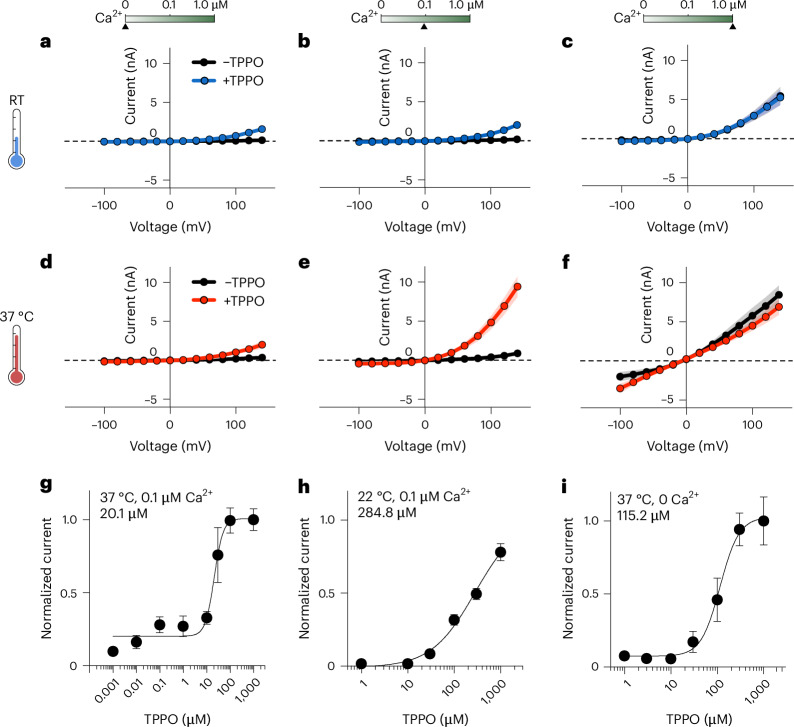
Three-way synergy between TPPO, temperature and Ca^2+^. **a**–**f**, Whole-cell currents measured in tsA cells overexpressing wild-type TRPM4 at 22 °C (room temperature, RT) (**a**–**c**) and 37 °C (**d**–**f**), with free intracellular Ca^2+^ concentrations of 0 µM (**a**,**d**), 0.1 µM (**b**,**e**) and 1 µM (**c**,**f**), in the absence or presence of 50 µM extracellular TPPO. A voltage protocol was applied, stepping from −100 mV to +140 mV in 20-mV increments, with each step lasting 100 ms. The currents from all measured cells were then averaged and plotted versus voltage to generate the mean current–voltage (*I*–*V*) curve, with the line representing the mean and the shaded area indicating the s.e.m. *n* values (the numbers of independently measured cells) are as follows: **a**, *n* = 9 (–TPPO), 9 (+TPPO); **b**, *n* = 7 (–TPPO), 8 (+TPPO); **c**, *n* = 9 (–TPPO), 9 (+TPPO); **d**, *n* = 7 (–TPPO), 10 (+TPPO); **e**, *n* = 8 (–TPPO), 8 (+TPPO); **f**, *n* = 6 (–TPPO), 6 (+TPPO). **g**–**i**, TPPO dose–response measurements for wild-type TRPM4 with 0.1 µM free intracellular Ca^2+^ at 37 °C (**g**), 0.1 µM Ca^2+^ at 22 °C (**h**) and 0 µM Ca^2+^ at 37 °C (**i**). A voltage protocol was applied every 10 s to monitor current changes: –100 mV for 200 ms, followed by +100 mV for 200 ms. Once the current was stabilized at each TPPO concentration, the current amplitudes at +100 mV from all measured cells were averaged, and the mean amplitudes were plotted and fitted to obtain the EC_50_. Current amplitudes were normalized either to the mean at 1,000 µM TPPO (**g**,**i**) or to the fitted maximal current (*I*_max_) from the Hill equation (**h**). Data are presented as mean ± s.e.m. *n* values are as follows: **g**, *n* = 7 for all concentrations; **h**, *n* = 5 at 1 µM and *n* = 7 for all other concentrations; **i**, *n* = 7 for all concentrations. Source data

**Fig. 2:**
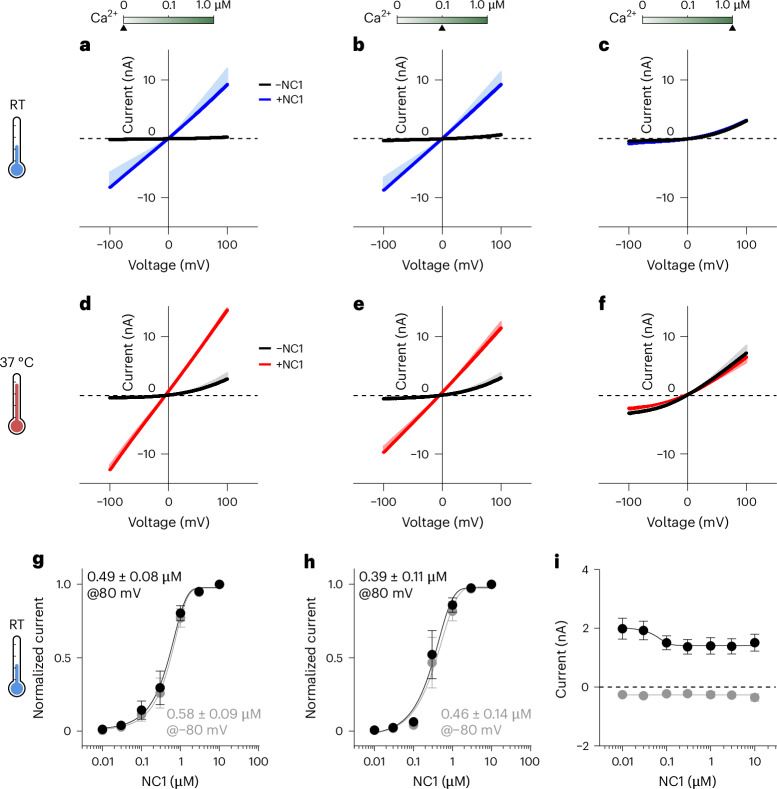
Ca^2+^-dependent gating reversal of NC1. **a**–**f**, Whole-cell currents measured in tsA cells overexpressing wild-type TRPM4 at 22 °C (**a**–**c**) and 37 °C (**d**–**f**), with free intracellular Ca^2+^ concentrations of 0 µM (**a**,**d**), 0.1 µM (**b**,**e**) and 1 µM (**c**,**f**). A voltage protocol was applied every 5 s to monitor current changes until steady state was reached: −100 mV for 50 ms, ramped to +100 mV over 200 ms and held at +100 mV for 50 ms. The currents with and without 1 µM extracellular NC1 in each condition were measured in a single cell. The currents from all measured cells were then averaged and plotted versus voltage to generate the mean *I*–*V* curve, with the line representing the mean and the shaded region indicating the s.e.m. For clarity, the s.e.m. envelope was plotted in one direction. *n* values are as follows: **a**, *n* = 5; **b**, *n* = 7; **c**, *n* = 5; **d**, *n* = 5; **e**, *n* = 4; **f**, *n* = 4. **g**–**i**, NC1 dose–response measurements for wild-type TRPM4 at 22 °C with 0 µM free intracellular Ca^2+^ (**g**), 0.1 µM Ca^2+^ (**h**) and 1 µM Ca^2+^ (**i**). A voltage protocol was applied every 5 s to monitor current changes: 0 mV for 50 ms, switch to +80 mV for 50 ms, followed by −80 mV for 50 ms and return to 0 mV for 50 ms. Once the current stabilized at each NC1 concentration, the current amplitudes at +80 mV and −80 mV were normalized to those at 10 µM NC1. Normalized currents from all measured cells were averaged and plotted as mean ± s.e.m. EC_50_ values obtained from individual cells were averaged to yield the final EC_50_ reported as mean ± s.e.m. (**g**,**h**). Data are presented as mean ± s.e.m. *n* values are as follows: **g**, *n* = 5; **h**, *n* = 5; **i**, *n* = 4. Source data

**Extended Data Fig. 1:**
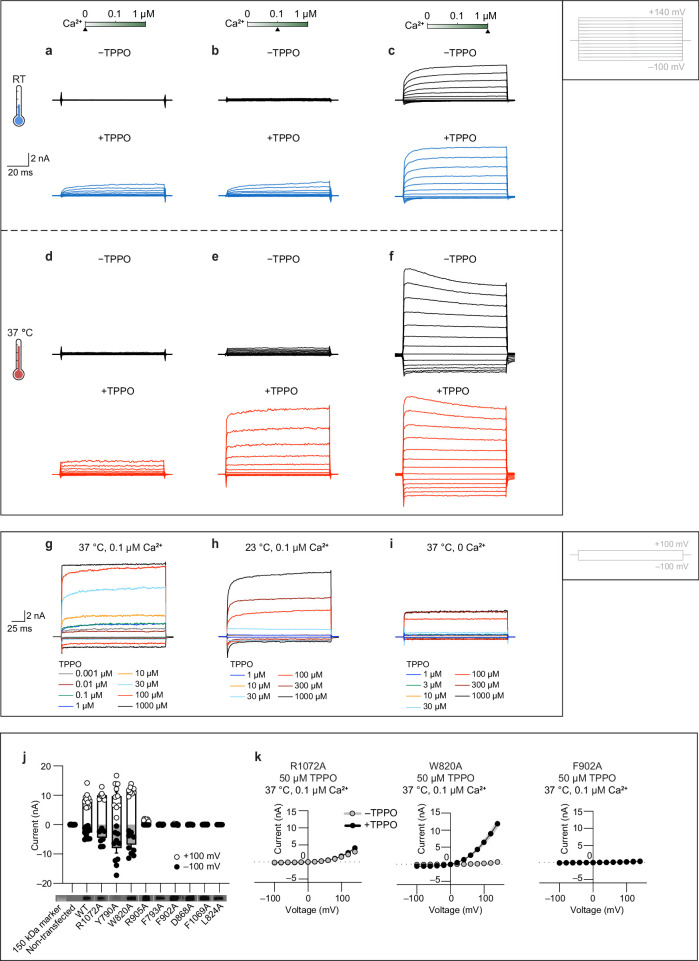
Electrophysiological characterization of TPPO-dependent activation of TRPM4. **a–f**, Representative whole-cell current traces recorded at room temperature (22 °C) and 37 °C with free intracellular Ca^2+^ concentrations of 0 µM (**a,d**), 0.1 µM (**b,e**), and 1 µM (**c,f**), evoked by a voltage-step protocol from –100 mV to +140 mV in 20-mV increments (100 ms per step). The currents without and with 50 µM extracellular TPPO at each condition were measured from a single cell. **g–i**, Representative whole-cell current traces from the TPPO dose response recordings acquired using a two-step protocol (–100 mV followed by +100 mV for 200 ms each) under 0.1 µM Ca^2+^ at 37 °C (**g**), 0.1 µM Ca^2+^ at 22 °C (**h**), and 0 µM Ca^2+^ at 37 °C (**i**). **j**, Whole-cell currents of non-transfected tsA cells and tsA cells overexpressing wild-type TRPM4 and TPPO binding-site mutants evoked by 1 μM free intracellular Ca^2+^ and measured at +100 and −100 mV at room temperature. Each point represents an independently measured cell, with bars indicate the mean. The numbers of independently measured cells are 8 (non-transfected tsA201), 10 (WT), 10 (R1072A), 7 (Y790A), 10 (W820A), 9 (W828A), 12 (S789A), 7 (R905A), 12 (F793A), 6 (F902A), 5 (D868A), 3 (E898A), 4 (F1069A), and 5 (L824A). Cell surface expression of wild-type TRPM4 and mutants is shown, confirming proper trafficking to the plasma membrane; uncropped gels are provided in Supplementary Fig. 2. **k**, Whole-cell currents measured in tsA cells overexpressing the R1072A, W820A and F902A mutants at 37 °C, with 0.1 µM free intracellular Ca^2+^ in the absence or presence of 50 µM extracellular TPPO. A voltage protocol stepping from −100 mV to +140 mV in 20-mV increments was applied, with each voltage step lasting 100 ms. The currents from all measured cells were then averaged and plotted versus voltage to generate the mean I–V curve, with the line representing the mean and the shaded indicating the s.e.m. The numbers of independently measured cells (n) are as follows: R1072A: n = 9 ( − TPPO) / 10 ( + TPPO); W820A: n = 10 ( − TPPO) / 12 ( + TPPO); F902A: n = 8 ( − TPPO) / 8 ( + TPPO)). Source data

**Extended Data Fig. 2:**
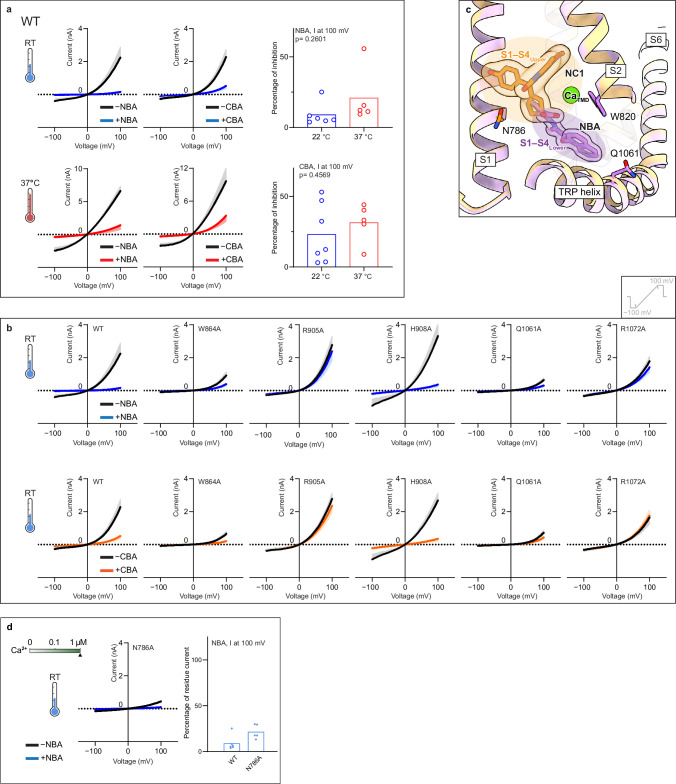
Electrophysiological characterization of TRPM4 inhibition by NBA and CBA. **a**, Left and middle panels show whole-cell currents measured in tsA cells overexpressing wild-type TRPM4 at 22 °C and 37 °C, with 1 µM free intracellular Ca^2+^. A voltage protocol was applied every 5 s to monitor current changes until reaching steady state: −100 mV for 50 ms, ramped to +100 mV over 200 ms, and held at +100 mV for 50 ms. The currents without and with 10 µM extracellular NBA or CBA at each condition were measured from a single cell. The currents from all measured cells were then averaged and plotted versus voltage to generate the mean I–V curve, with the line representing the mean and the shaded indicating the s.e.m. For clarity, the s.e.m. envelope was plotted in one direction. The numbers of independently measured cells are as follows: 6 (NBA, 22 °C), 5 (NBA, 37 °C), 7 (CBA, 22 °C), and 5 (CBA, 37 °C). Right panels show quantification of CBA- and NBA-dependent inhibition at +100 mV for the recordings shown in the left and middle panels. The percentage of remaining currents after the application of NBA or CBA was calculated by dividing the steady-state current recorded after NBA or CBA application by the steady-state current recorded before application. Each point represents an independently measured cell, and bars indicate the mean. A t-test was used to compare inhibition at 22 °C versus 37 °C. A two-sided t-test was performed, with p-values of 0.2601 (NBA) and 0.4569 (CBA). **b**, Whole-cell currents measured in tsA cells overexpressing wild-type TRPM4 or NBA/CBA binding-site mutants at 22 °C, with 1 µM free intracellular Ca^2+^, without and with 10 µM extracellular NBA or CBA. A voltage protocol was applied every 5 s to monitor current changes until reaching steady state: −100 mV for 50 ms, ramped to +100 mV over 200 ms, and held at +100 mV for 50 ms. The currents without and with 10 µM extracellular NBA or CBA at each condition were measured from a single cell. The currents from all measured cells were then averaged and plotted versus voltage to generate the mean I–V curve, with the line representing the mean and the shaded indicating the s.e.m. For clarity, the s.e.m. envelope was plotted in one direction. Measurements and analysis were performed as in panel (**a**). The numbers of independently measured cells (n) are as follows. NBA: n = 6 (WT), n = 6 (W864A), n = 5 (R905A), n = 5 (H908A), n = 6 (Q1061A), n = 6 (R1072A); CBA: n = 7 (WT), n = 6 (W864A), n = 5 (R905A), n = 5 (H908A), n = 5 (Q1061A), n = 4 (R1072A). **c**, Structural comparison of the Ca^2+^/NBA-bound state (purple, PDB-ID 9Z26) and the NC1-bound state (orange, PDB-ID 9Z21) of TRPM4, superimposed using the S1–S4 domain (residues 760–910). NBA and NC1 are shown in stick representation with a transparent envelope, Ca_TMD_ as a green sphere and the protein backbone in cartoon representation and N786 side chain in stick representation. The filled orange and blue ellipses mark the upper and lower pockets within the S1–S4 domain, respectively. **d**, Left panel shows whole-cell currents measured in tsA cells overexpressing the N786A mutant at 22 °C, with 1 µM free intracellular Ca^2+^, without and with 10 µM extracellular NBA in the same cells, averaged from 5 independent cells. Right panel shows quantification of NBA-dependent inhibition at +100 mV for wild-type TRPM4 and the N786A mutant. Measurements and analysis were performed as in panel (**a**). Each point represents an independently measured cell, and bars indicate the mean. The numbers of independently measured cells are 6 (WT), and 5 (N786A). A two-sided t-test was performed, with a p-value of 0.0272. Source data

Using patch–clamp electrophysiology, we tested these compounds at both room temperature and 37 °C. Under previously reported assay conditions—room temperature with either no free intracellular Ca^2+^ or basal levels of free intracellular Ca^2+^ (100 nM)—we reproduced established results: 50 μM TPPO showed negligible activity on TRPM4, comparable to the control group, for which basal Ca^2+^ levels alone are insufficient to drive channel activation (Fig. 1a,b and Extended Data Fig. 1a,b); NC1 activated the channel, eliciting largely linear, voltage-independent currents (Fig. 2a,b); and NBA and CBA produced robust inhibition of Ca^2+^-induced TRPM4 current (Extended Data Fig. 2a). Strikingly, elevating the temperature to 37 °C revealed a hidden pharmacological behavior. Although NC1, NBA and CBA retained their room-temperature profiles (Fig. 2d,e and Extended Data Fig. 2a), TPPO displayed a completely different response—robustly activating TRPM4 currents (Fig. 1e and Extended Data Fig. 1e). These observations highlight a critical gap in conventional pharmacological screening: assays performed under non-physiological conditions could both overlook potential active compounds and misclassify ligand selectivity.

### Ca^2+^ as a key determinant of agonist recognition and potency in TRPM4

Ca^2+^ is the only known endogenous agonist of TRPM4 and, together with temperature, drives the transition from its cold to warm conformations^10^. Building on our findings that temperature reshapes TRPM4 pharmacology (Fig. 1e), we next explored whether Ca^2+^ modulates the binding and potency of the exogenous activators TPPO and NC1.

Under Ca^2+^-free conditions, achieved through EGTA buffering, 50 µM TPPO produced negligible activation of TRPM4 (Fig. 1d and Extended Data Fig. 1d). However, the addition of 100 nM free intracellular Ca^2+^ led to robust activation (Fig. 1e, red traces, and Extended Data Fig. 1e). Thus, even basal Ca^2+^ concentrations—although insufficient to open the channel on their own (Fig. 1e, black trace, and Extended Data Fig. 1e)—markedly enhance TPPO-induced TRPM4 activation. To quantify this apparent synergy, we determined TPPO dose–response relationships. Under physiological temperature, TPPO potency increased by ~14-fold under basal Ca^2+^ (Fig. 1g,h and Extended Data Fig. 1g,h), whereas basal Ca^2+^ enhanced TPPO potency by ~6-fold at 37 °C (Fig. 1g,i and Extended Data Fig. 1g,i). In the absence of both Ca^2+^ and physiological temperature, TPPO weakly activated TRPM4 at submillimolar concentrations, failing to reach saturation even at millimolar levels. When intracellular Ca^2+^ rose to an activating concentration (1 µM), the effect of TPPO was largely—although not completely—masked. Under these conditions, the current recorded in the presence of TPPO was relatively linear and closely resembled that observed without TPPO, a phenotype we previously reported for TRPM4 activated by 1 µM Ca^2+^ at 37 °C (ref. ^10^), with only minor differences that might reflect a combined contribution of TPPO and Ca^2+^. Together, these observations are consistent with TPPO and Ca^2+^ acting through a shared activation pathway, with Ca^2+^ becoming the dominant determinant of the channel behavior at high concentrations (Fig. 1c,f and Extended Data Fig. 1c,f).

These data collectively demonstrate that physiological context is essential for TPPO efficacy, revealing a three-way synergy among temperature, Ca^2+^ and ligand binding. This dependence overturns the view that TPPO is inactive toward TRPM4, revealing instead that it potently activates the channel—opposite to its inhibitory action on the closely related TRPM5 (ref. ^35^). This finding underscores how ligand behavior can be fundamentally mischaracterized when assays are performed under non-physiological conditions.

**Extended Data Fig. 3:**
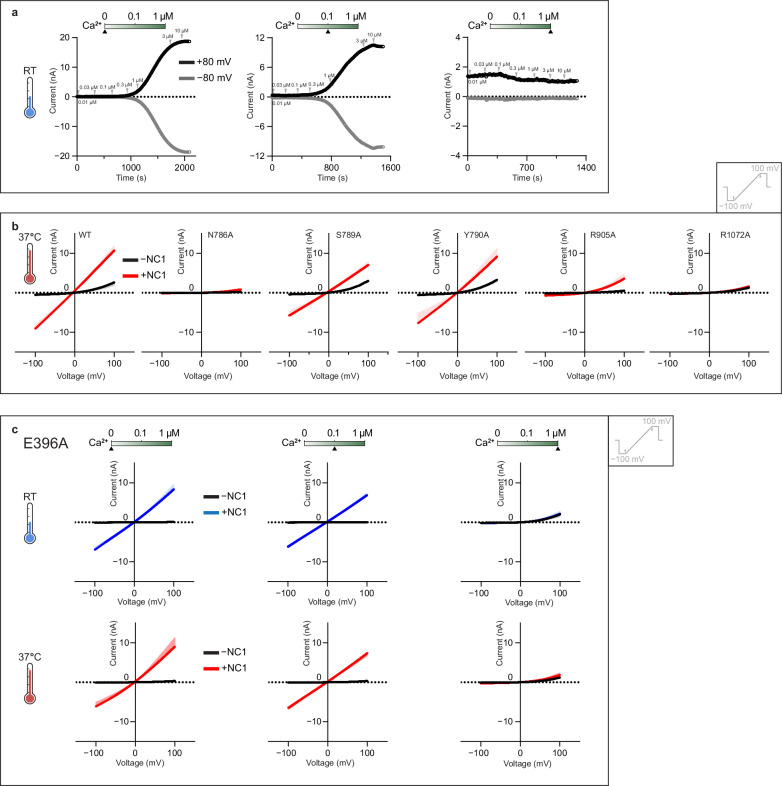
Electrophysiological characterization of NC1-dependent activation of TRPM4. **a**, The representative current traces for the NC1 dose-response measurements for wild-type TRPM4 at 22 °C with free intracellular Ca^2+^ of 0 µM, 0.1 µM, and 1 µM. A voltage protocol was applied every 5 s to monitor current changes: 0 mV for 50 ms, switch to +80 mV for 50 ms, followed by −80 mV for 50 ms, and return to 0 mV for 50 ms. **b**, Whole-cell currents measured in tsA cells overexpressing wild-type TRPM4 or NC1 binding-site mutants at 37 °C, with 0.1 µM free intracellular Ca^2+^, without and with 1 μM extracellular NC1. A voltage protocol was applied every 5 s to monitor current changes until reaching steady state: −100 mV for 50 ms, ramped to +100 mV over 200 ms, and held at +100 mV for 50 ms. The currents without and with NC1 at each condition were measured from a single cell. The currents from all measured cells were then averaged and plotted versus voltage to generate the mean I–V curve, with the line representing the mean and the shaded indicating the s.e.m. For clarity, the s.e.m. envelope was plotted in one direction. The numbers of independently measured cells are 5 (WT), 5 (N786A), 4 (S789A), 5 (Y790A), 5 (R905A), and 4 (R1072A). **c**, Whole-cell currents were measured in tsA cells overexpressing the E396A mutant of TRPM4 at 22 °C (upper panels) and 37 °C (lower panels), with intracellular free Ca^2+^ concentrations of 0 µM, 0.1 µM, and 1 µM. A voltage protocol was applied every 5 s to monitor current changes: −100 mV for 50 ms, ramped to +100 mV over 200 ms and held at +100 mV for 50 ms. The current without or with 1 μM extracellular NC1 in each condition was measured in a single cell. The currents from all measured cells were then averaged and plotted versus voltage to generate the mean I–V curve, with the line representing the mean and the shaded indicating the s.e.m. For clarity, the s.e.m. envelope was plotted in one direction. The numbers of independently measured cells are 5 (0 µM Ca^2+^, 22 °C), 5 (0.1 µM Ca^2+^, 22 °C), 5 (1 µM Ca^2+^, 22 °C), 5 (0 µM Ca^2+^, 37 °C), 5 (0.1 µM Ca^2+^, 37 °C), and 5 (1 µM Ca^2+^, 37 °C). Source data

We next examined whether Ca^2+^ influences NC1, previously reported as the first Ca^2+^-independent agonist of TRPM4 (ref. ^36^). Under Ca^2+^-free and 100 nM Ca^2+^ conditions, NC1 evoked nearly identical currents with linear current–voltage relationships, distinct from Ca^2+^-activated currents. This confirms that NC1 can activate TRPM4 independently, with a half-maximal effective concentration (EC_50_) in the submicromolar range (Fig. 2a,b,d,e,g,h and Extended Data Fig. 3a). However, at 1 µM Ca^2+^, a physiologically relevant level during cellular stress that robustly activates TRPM4, the response changed dramatically, becoming strongly outwardly rectified (Fig. 2c,f)—a hallmark of canonical Ca^2+^-mediated activation^14^. This striking reversal suggests that NC1 is not simply Ca^2+^-independent: once Ca^2+^ reaches activating levels, its action dominates, functionally masking NC1-mediated gating even at saturating NC1 concentrations (Fig. 2i). Thus, rather than bypassing the Ca^2+^ requirement, NC1 displays biphasic Ca^2+^ dependence: it is permissive at basal Ca^2+^ levels, yet exhibits little to no additional effect at higher Ca^2+^ concentrations. This behavior is most consistent with NC1 becoming functionally masked or antagonized at elevated Ca^2+^ levels, either through reduced or abolished NC1 binding due to Ca^2+^-dependent competition or through allosteric coupling between NC1- and Ca^2+^-binding sites in scenarios in which co-binding occurs. Which mechanism is more likely is discussed below (see ‘Mechanistic basis for Ca^2+^-dependent gating reversal of NC1’).

Taken together, these results reveal distinct modes of Ca^2+^–agonist interplay in TRPM4. TPPO requires basal Ca^2+^ (and temperature) to act effectively on the channel, whereas NC1 functions independently of Ca^2+^ at rest but loses efficacy once Ca^2+^ rises to stimulatory levels. This contrast illustrates how the same intrinsic cofactor can differentially tune ligand action through cooperative or antagonistic coupling. More broadly, they reinforce the importance of evaluating ion-channel pharmacology under physiological conditions, as environmental context can fundamentally alter both efficacy and apparent selectivity.

### Structural basis of the three-way synergy between TPPO, temperature and Ca^2+^

**Extended Data Fig. 4:**
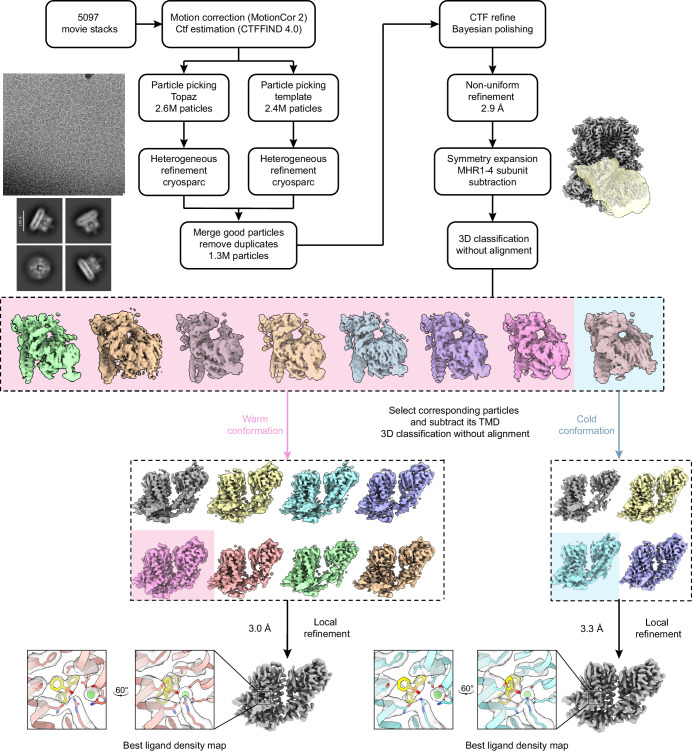
Cryo-EM data processing workflow for the Ca^2+^/TPPO–TRPM4–37 °C dataset. A representative micrograph (out of 5097 micrographs) and representative 2D classification images are shown. The final consensus map and the TMD-focused map with the best-resolved ligand density are displayed with their respective resolutions.

**Extended Data Fig. 5:**
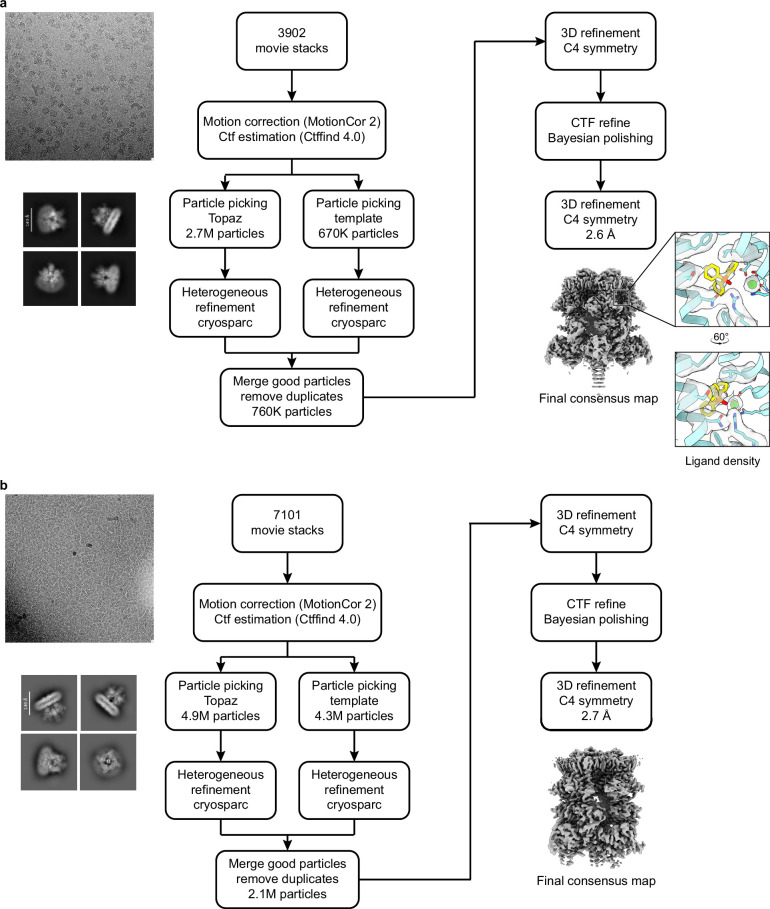
Cryo-EM data processing workflow for the Ca^2+^/TPPO–TRPM4–18 °C (a) and EGTA/TPPO–TRPM4–37 °C (b) datasets. A representative micrograph for Ca^2+^/TPPO–TRPM4–18 °C (**a**, out of 3092 micrographs) and EGTA/TPPO–TRPM4–37 °C (**b**, out of 7101 micrographs), and representative 2D classification images are shown. The final maps are displayed with their respective resolutions.

To elucidate how temperature and Ca^2+^ shape TPPO activation of TRPM4, we determined cryo-EM structures of the channel in the presence of TPPO at 18 °C and 37 °C, under both Ca^2+^-free and Ca^2+^-saturated conditions. All the structures were resolved to high quality at ~2.5- to 2.9-Å resolutions, allowing unambiguous identification of ligand-binding sites (Extended Data Figs. 4 and 5, Supplementary Fig. 1 and Supplementary Table 1).

**Fig. 3:**
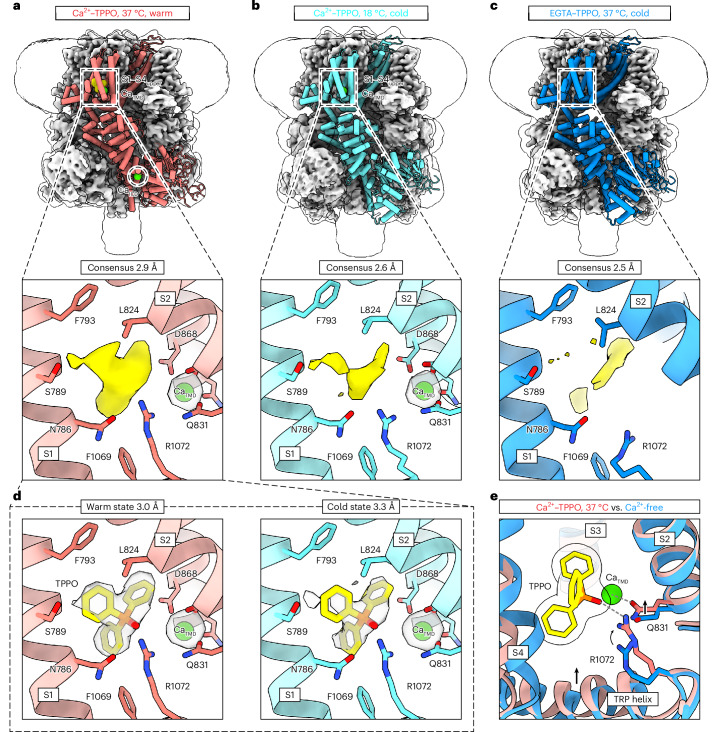
Structural basis of the three-way synergy between TPPO, temperature and Ca^2+^. **a**–**c**, Structures of TRPM4 in complex with Ca^2+^–TPPO at 37 °C (**a**, PDB 9Z1W), Ca^2+^–TPPO at 18 °C (**b**, PDB 9Z1Z) and EGTA–TPPO at 37 °C (**c**, PDB 9Z20). One subunit is shown in cartoon representation, and the remaining three subunits are displayed as a cryo-EM map, viewed parallel to the membrane. The transparent envelope outlines the detergent micelle and the disordered region of the carboxy-terminal coiled-coil. TPPO is highlighted in yellow, and Ca^2+^ in green. The resolution of the consensus map is indicated below. Bottom: the details of the S1–S4 binding site in the consensus maps. The protein backbone is shown in cartoon representation, with key interacting residues in stick representation. Densities at the TPPO binding site (yellow) and at the Ca_TMD_ site (transparent in **a** and **b**) are contoured at 4.5 *σ*, where *σ* denotes the standard deviation of the map density values. **d**, Details of the S1–S4 binding site in the warm (left, PDB 9Z1X) and cold (right, PDB 9Z1Y) states, obtained from classification of the Ca^2+^–TPPO dataset collected at 37 °C. The resolution of each cryo-EM map is indicated. Densities at the TPPO binding site and the Ca_TMD_ site are shown in transparent surface representations contoured at 9 *σ*. The fitted TPPO molecule is shown in stick representation, and Ca_TMD_ as a green sphere. **e**, Structural comparison of TRPM4 with Ca^2+^–TPPO at 37 °C (salmon, PDB 9Z1X) and the Ca^2+^-free apo state (blue, PDB 9B93), superimposed using the S1–S4 (residues 760–910). TPPO is shown in stick representation with a transparent envelope, and Ca_TMD_ as a green sphere. Black arrows indicate the approximate direction of movement of key residues and the TRP helix. The dashed lines mark key interactions.

In the consensus map of the Ca^2+^–TPPO–TRPM4 (37 °C) dataset, we observed a prominent tripod-shaped density in the S1–S4 domain of each protomer. This feature closely matches the shape of TPPO and is located directly above the Ca_TMD_ site (Fig. 3a). We therefore designated this pocket as the S1–S4_upper_ site, which spatially overlaps with the previously identified deacetyl bisacodyl binding pocket^25^. Subunit-level classification revealed that warm and cold conformations coexist in this dataset, with the warm state predominating (Extended Data Fig. 4). The ligand density is markedly stronger in the warm state, whereas a weaker yet still discernible density is present in the cold state (Fig. 3d; this minor cold state is discussed at the end of this section). In this pocket, TPPO occupies a hydrophobic cavity and forms a critical polar interaction between its phosphoryl moiety and R1072 on the TRP helix (Fig. 3d, left). To confirm this interaction, we mutated key coordinating residues to alanine and conducted electrophysiological recordings. Most substitutions impaired Ca^2+^-dependent activation, likely by disrupting the integrity of the adjacent Ca_TMD_ site, making it impossible to unambiguously isolate their effects on TPPO, because TPPO requires Ca_TMD_ for its activity to manifest (Extended Data Fig. 1j). By contrast, R1072A preserved Ca^2+^-evoked gating yet abolished TPPO-dependent activation, thereby confirming the functional relevance of the TPPO-binding site (Extended Data Fig. 1k).

In the Ca^2+^-bound cold conformation from the Ca^2+^–TPPO–TRPM4 (18 °C) dataset, only a faint and incomplete signal was discernible at the S1–S4_upper_ site at very low contour levels (Fig. 3b). The density formed a triangular outline reminiscent of TPPO but was markedly smaller than the full ligand, consistent with partial or unstable occupancy. In the Ca^2+^-free cold conformation from the EGTA–TPPO–TRPM4 (37 °C) dataset, this feature disappeared entirely, with no trace of TPPO density detectable (Fig. 3c).

Thus, TPPO binding exhibits a clear dependence on the cold–warm conformational equilibrium of TRPM4, a transition that is cooperatively governed by temperature and Ca^2+^ concentration^10^. This structural observation coincides with our electrophysiological finding that TPPO, temperature and Ca^2+^ synergize to promote channel activation (Fig. 1a,b,d,e). Together, these results suggest that TPPO efficacy is tightly coupled to the conformational landscape defined by these two intrinsic modulators.

To dissect the mechanism underlying this coupling, we compared the Ca^2+^-free cold and Ca^2+^–TPPO-bound warm structures. In the Ca^2+^-free cold conformation, R1072 is displaced from the S1–S4_upper_ pocket and stabilized by an interaction with Q831 on S2—a key residue involved in Ca_TMD_ binding—thereby precluding TPPO binding (Fig. 3e). After transitioning to the warm state, upward movement of the intracellular domain tilts the TRP helix, moving R1072 into position for TPPO coordination (Fig. 3e). Concurrently, Ca^2+^ binding at the Ca_TMD_ site recruits Q831 for Ca^2+^ coordination, releasing R1072 from its locked pose for direct interaction with the TPPO phosphoryl group (Fig. 3e). Together, these coupled rearrangements prime the S1–S4_upper_ pocket for effective ligand engagement.

**Extended Data Fig. 6:**
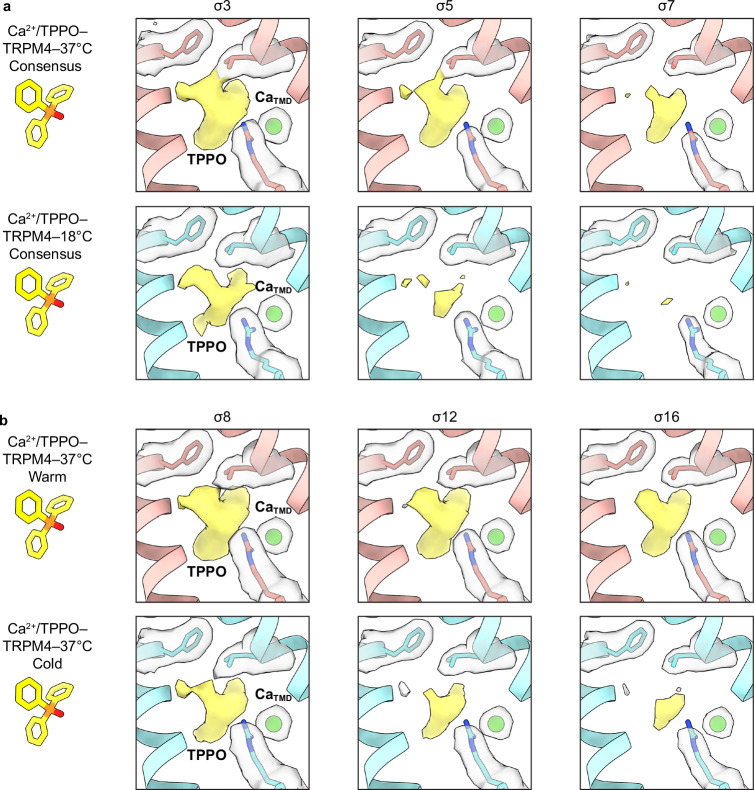
Densities of the TPPO binding site from various datasets or conformations contoured at different σ levels. **a,b,** Densities at the TPPO binding site are shown as yellow surfaces. For comparison, densities for Ca_TMD_ and surrounding interacting residues are displayed as transparent surfaces. The TPPO molecule in the pose fitted to the Ca^2+^/TPPO–TRPM4–37 °C warm map is shown on the left in stick representation.

Notably, a weak TPPO-shaped density was also detected in the minor Ca^2+^-bound cold conformation in the Ca^2+^–TPPO–TRPM4 (37 °C) dataset, identified through single-subunit classification (Extended Data Fig. 4). This density was weaker than that in the warm conformation, but stronger than in the corresponding Ca^2+^-bound cold conformation resolved at 18 °C (Fig. 3b, bottom, Fig. 3d and Extended Data Fig. 6), indicating that TPPO occupancy increases with temperature, even in the same overall protein conformation. Because the backbone architecture of the binding site remains nearly identical between the two cold structures, this enhancement likely arises from subtle, temperature-dependent side-chain rearrangements—below the resolution of our maps (~2.6 Å local)—that fine-tune ligand packing and render the interaction thermodynamically more favorable.

Together, these results reveal that TPPO selectively engages the warm conformation of TRPM4 by exploiting temperature- and Ca^2+^-dependent changes in local binding energetics. This provides a direct structural basis for the observed three-way synergy, illustrating how intrinsic modulators reshape the binding pocket to control ligand efficacy at the atomic level.

### Mechanistic basis for Ca^2+^-dependent gating reversal of NC1

**Extended Data Fig. 7:**
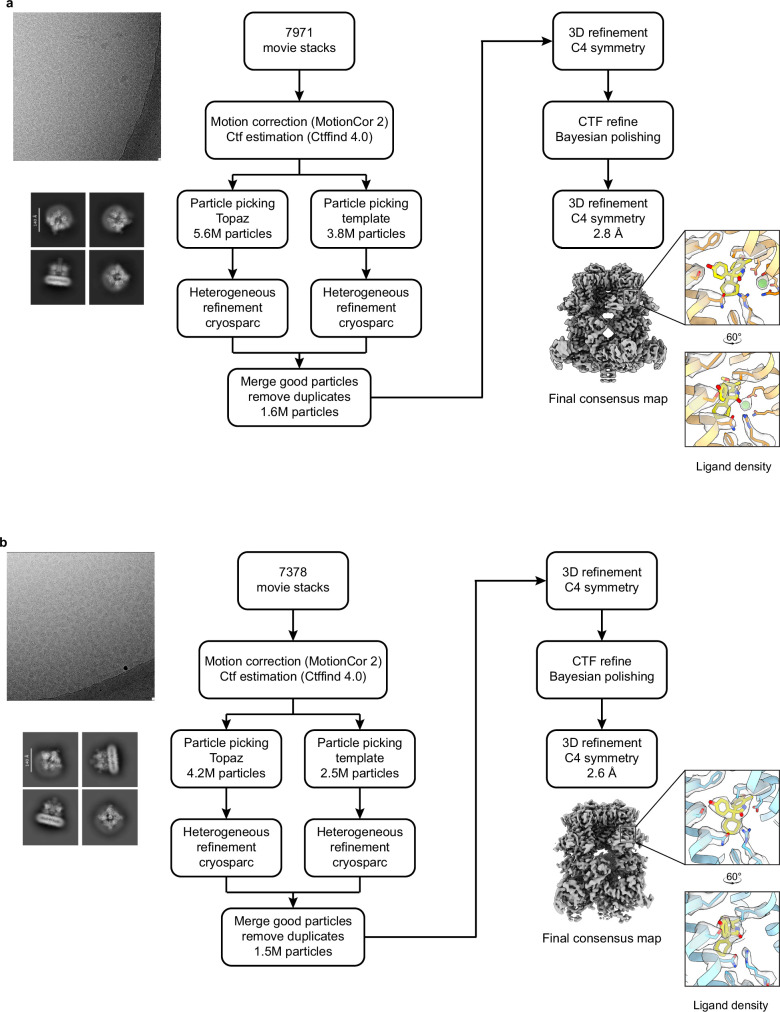
Cryo-EM data processing workflow for the Ca^2+^/NC1–TRPM4–37 °C (a) and EGTA/NC1–TRPM4–37 °C (b) datasets. A representative micrograph for Ca^2+^/NC1–TRPM4–37 °C (**a**, out of 7971 micrographs) and EGTA/NC1–TRPM4–37 °C (**b**, out of 7378 micrographs), and representative 2D classification images are shown. The final maps are displayed with their respective resolutions.

Building on our observation that NC1 activates TRPM4 in the absence of Ca^2+^ but loses efficacy once Ca^2+^ reaches activating levels (Fig. 2a–f), we sought to define the structural basis of this gating reversal. To this end, we determined cryo-EM structures of TRPM4 in complex with NC1 at 37 °C under Ca^2+^-free and Ca^2+^-bound conditions, resolved to 2.6 and 2.8 Å, respectively (Extended Data Fig. 7, Supplementary Fig. 1 and Supplementary Table 2).

**Fig. 4:**
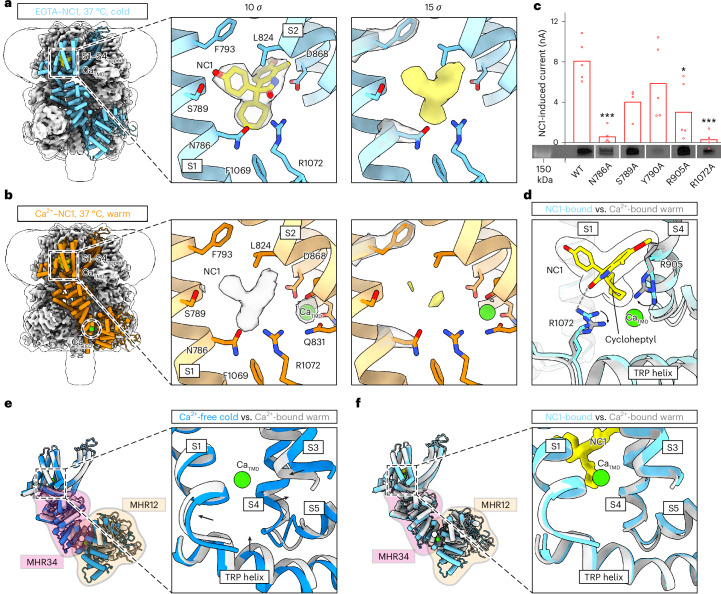
Mechanistic basis for Ca^2+^-dependent gating reversal of NC1. **a**,**b**, Left: the structures of TRPM4 in complex with EGTA and NC1 at 37 °C (**a**, PDB 9Z22) and Ca^2+^ and NC1 at 37 °C (**b**, PDB 9Z21). One subunit is shown in cartoon representation, and the remaining three subunits are displayed as a cryo-EM map, viewed parallel to the membrane. The transparent envelope outlines the detergent micelle and the disordered region of the C-terminal coiled-coil. Middle: the details of the S1–S4 binding site, with the protein backbone in cartoon representation and key interacting residues in stick representation. Densities at the NC1 binding site, and at the Ca_TMD_ site (only in **b**), are shown as transparent surface representations contoured at 10 *σ*. The fitted NC1 molecule in (only in **a**) is shown in stick representation, and Ca_TMD_ as a green sphere (only in **b**). Right: the same regions as in the middle panels, but densities for well-defined side chains in the binding site are also shown, contoured at 15 *σ*. **c**, NC1-induced whole-cell current amplitudes at +100 mV in cells overexpressing wild-type TRPM4 and binding-site mutants, calculated by subtracting the steady-state current recorded before NC1 application from that recorded after application. Each point represents an independently measured cell, with bars indicating the mean. *n* values are 5 (WT), 5 (N786A), 4 (S789A), 5 (Y790A), 5 (R905A) and 4 (R1072A). Statistical analysis was performed using one-way ANOVA with Bonferroni’s post hoc test, comparing each mutant with the wild type (*P < 0.05, ***P < 0.001). The adjusted *P* values for each mutant compared with the wild-type (WT) control were 0.0002 for N786A, 0.0741 for S789A, 0.7002 for Y790A, 0.0103 for R905A and 0.0002 for R1072A. Averaged raw data are shown in Extended Data Fig. 3b. Cell-surface expression of wild-type TRPM4 and mutants is shown, confirming proper trafficking to the plasma membrane; uncropped gels are provided in Supplementary Fig. 2. **d**, Structural comparison of the NC1-bound state (cyan, PDB 9Z22) and the Ca^2+^-bound warm state (blue, PDB 9B8W) of TRPM4, superimposed using the S1–S4 (residues 760–910). NC1 is shown in stick representation with a transparent envelope, and Ca_TMD_ as a green sphere. Black arrows indicate the approximate direction of movement of key residues. The dashed line marks a key interaction. **e**, Structural comparison of a single subunit of the Ca^2+^-free cold state (blue, PDB 9B93) and the Ca^2+^-bound warm state (gray, PDB 9B8W) of TRPM4, superimposed using the S1–S4 (residues 760–910). MHR3 and MHR4 are outlined with a pink envelope, and MHR1 and MHR2 with an orange envelope. Right: details of the S1–S4 binding site and its surroundings. Ca_TMD_ is shown as a green sphere, and the black arrows indicate the movement of structural elements forming the binding site upon Ca^2+^ binding. **f**, Structural comparison of the NC1-bound state (cyan, PDB 9Z22) and the Ca^2+^-bound warm state (gray, PDB 9B8W) of TRPM4. The structures are superimposed and displayed similarly to how they are shown in **e**. NC1 is shown in stick representation, and Ca_TMD_ as a green sphere. Source data

In the Ca^2+^-free dataset, a well-defined tripod-shaped density was observed in the S1–S4 domain (~2.6 Å local resolution) that occupied the same pocket as the TPPO- and deacetyl-bisacodyl-binding sites^25^; no Ca^2+^ density appeared at the Ca_TMD_ site, as expected (Fig. 4a). The density’s shape and dimensions clearly correspond to NC1. Although NC1 is larger than TPPO, it features a similar three-ring scaffold, with its indolinone carbonyl oxygen positioned analogously to TPPO’s phosphoryl oxygen (Fig. 3d, left, and Fig. 4a). Consequently, NC1 binds in a similar pose to TPPO, engaging an equivalent network of residues, including a key polar contact between its indolinone carbonyl oxygen and R1072 on the TRP helix (Fig. 4a). In line with this, alanine substitution of R1072 and other residues in the binding site abolished or markedly reduced NC1-mediated activation (Fig. 4c and Extended Data Fig. 3b), confirming that NC1, like TPPO, binds to the S1–S4_upper_ site.

Notably, in the Ca^2+^-bound dataset, NC1 density at the same S1–S4_upper_ pocket was markedly weaker (Fig. 4b). This diminished occupancy aligns with our electrophysiological findings, in which 1 µM Ca^2+^ strongly suppressed the linear, NC1-evoked currents characteristic of Ca^2+^-free conditions and restored the canonical Ca^2+^-dependent gating pattern (Fig. 2a–f). Together, these data indicate that NC1 binding is strongly disfavored in the presence of Ca^2+^. Although we cannot exclude the possibility of NC1 and Ca^2+^ cobinding to produce an electrophysiological profile similar to that of Ca^2+^ alone, the pronounced weakening of NC1 density in the Ca^2+^-bound structures suggests that a Ca^2+^-dependent reduction, or near abolition, of NC1 binding is the most likely explanation. Accordingly, we interpret the weak residual density as arising from the oversaturating NC1 concentration used for cryo-EM, rather than as arising from stable cobinding. Notably, the antagonism does not result from direct competition for a shared site, but instead from allosteric interference between the NC1 pocket and the Ca_TMD_ site (Fig. 4b).

To understand the basis of this interference, we compared the Ca^2+^-free NC1–TRPM4 and Ca^2+^–TRPM4 complexes. Two local structural changes accompany Ca^2+^ coordination at the Ca_TMD_. First, Ca^2+^ binding repositions the TRP helix, shifting R1072 away from the indolinone carbonyl oxygen that is central to NC1 recognition (Fig. 4d). Second, Ca^2+^ binding brings the positively charged R905 into close proximity with NC1’s hydrophobic seven-membered cycloheptyl ring, creating an electrostatically unfavorable juxtaposition that further destabilizes ligand binding (Fig. 4d). Together, these antagonistic rearrangements explain why NC1 affinity drops at increased Ca^2+^ concentration.

In summary, NC1 is the first example of a TRPM4 activator that is temperature-independent yet negatively regulated by intrinsic Ca^2+^. Structural and functional analyses together uncover a Ca^2+^-dependent gating reversal, whereby Ca^2+^ reshapes the allosteric network to displace a previously bound activator. This mechanism contrasts sharply with TPPO, whose efficacy requires Ca^2+^ synergy at physiological temperature, underscoring the opposing logic by which intrinsic modulators shape TRPM4 pharmacology.

### NC1 overrides Ca^2+^-dependent ICD–TMD allosteric regulation

The defining feature of NC1 is that it activates TRPM4 without requiring Ca^2+^ at either of the two regulatory sites (Fig. 2a,d): the agonist site, Ca_TMD_, and the temperature-dependent allosteric site in the intracellular domain (Ca_ICD_). In canonical gating, Ca^2+^ and temperature cooperatively couple the ICD and TMD to drive activation: Ca^2+^ binding at the temperature-sensitive Ca_ICD_ shifts the channel from the cold to the warm state, establishing the primed conformation in which Ca^2+^ binding at the Ca_TMD_ opens the pore at physiologically relevant membrane potentials^10^. To understand how NC1 circumvents this mechanism, we compared three structural states: Ca^2+^-free cold, Ca^2+^-bound warm and NC1-bound. The transition from the Ca^2+^-free cold state to the Ca^2+^-bound warm state involves major rearrangements in the ICD coupled to a substantial shift in the TRP helix and TMD (Fig. 4e). By contrast, in the NC1-bound structure, the ICD remains in the cold-like state, yet the TMD closely resembles the warm-state conformation even in the absence of Ca^2+^ at the Ca_TMD_ (Fig. 4f). These observations suggest that NC1 functionally substitutes for Ca^2+^ at the TMD while operating independently of Ca^2+^-dependent priming at the ICD.

Electrophysiology supported this model: NC1 elicited robust currents of comparable amplitude at both room and physiological temperatures (Fig. 2b,e)—in contrast to Ca^2+^-dependent TRPM4 activation, which is strongly enhanced at 37 °C through Ca_ICD_-mediated ICD–TMD coupling^10,15^. Thus, NC1 activates the channel without engaging the Ca_ICD_-driven allosteric control. To directly test this mechanism, we examined the E396A mutant, in which Ca_ICD_ binding is abolished and TRPM4 is locked in the cold-like conformation regardless of temperature^10^. Remarkably, NC1 still produced strong, temperature-independent activation in this mutant, confirming that NC1 does not require the ICD–TMD regulation (Extended Data Fig. 3c). This unprecedented mode of action reveals an alternative allosteric pathway in TRPM4 and highlights NC1 as a promising scaffold for therapeutic development, particularly under conditions in which intracellular Ca^2+^ fluctuates.

### NBA and CBA inhibit TRPM4 through the S1–S4_lower_ site

Pharmacological inhibition of TRPM4 is of broad clinical importance because channel overactivation contributes to arrhythmogenic cardiac disorders, ischemic injury and cancer progression^18,21,38^. However, our previous work revealed that the endogenous antagonist ATP markedly loses potency at physiological temperature because its binding location shifts between the cold and warm conformations of the channel^10^. This temperature sensitivity limits ATP’s ability to suppress TRPM4 under normal cellular conditions^39^.

To identify inhibitors that remain effective in the physiological conformational landscape, we turned to NBA and its analog CBA^37^. In contrast to ATP and other temperature-sensitive ligands, NBA and CBA inhibit TRPM4 in a temperature-independent manner, maintaining high potency at 37 °C (Extended Data Fig. 2a). This makes them ideal probes for uncovering how TRPM4 inhibition can be achieved under physiological conditions through a temperature-insensitive mechanism.

**Fig. 5:**
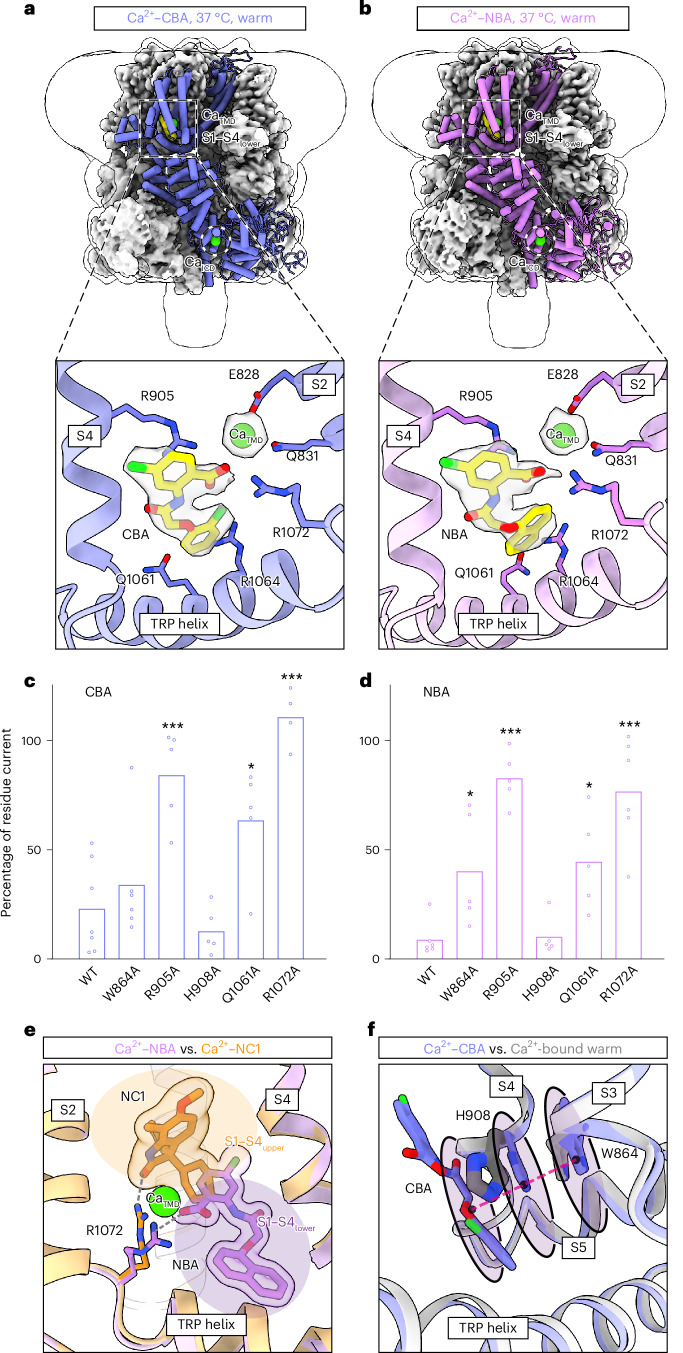
NBA and CBA inhibit TRPM4 through the S1–S4_lower_ site. **a**,**b**, Structure of TRPM4 in complex with Ca^2+^–CBA at 37 °C (**a**, PDB 9Z23) and Ca^2+^–NBA at 37 °C (**b**, PDB 9Z25). One subunit is shown in cartoon representation, and the remaining three subunits are displayed as a cryo-EM map, viewed parallel to the membrane. The transparent envelope outlines the detergent micelle and the disordered region of the C-terminal coiled-coil. CBA and NBA are highlighted in yellow, and Ca^2+^ in green. Bottom: the details of the S1–S4 binding site, with the protein backbone in cartoon representation and key interacting residues in stick representation. Densities for CBA and Ca_TMD_ (**a**) and for NBA and Ca_TMD_ (**b**) are shown in transparent surface representations contoured at 17 *σ* and 20 *σ*, respectively. The fitted CBA and NBA molecules are shown in stick representation, and Ca_TMD_ as a green sphere. **c**,**d**, CBA- and NBA-dependent inhibition at +100 mV in cells overexpressing wild-type TRPM4 and binding-site mutants. The percentage of remaining currents after the application of NBA or CBA at 22 °C was calculated by dividing the steady-state current recorded after the application by the steady-state current recorded before application. Each point represents an independently measured cell, with bars indicating the mean. *n* values are: NBA, 6 (WT), 5 (W864A), 5 (R905A), 5 (H908A), 5 (Q1061A) and 6 (R1072A); CBA, 7 (WT), 6 (W864A), 5 (R905A), 5 (H908A), 5 (Q1061A) and 4 (R1072A). Statistical analysis was performed using one-way ANOVA with Bonferroni’s post hoc test, comparing each mutant with wild type. *P < 0.05, ***P < 0.001. Adjusted *P* values for each mutant compared with the wild-type (WT) control were as follows: CBA, >0.9999 for W864A, 0.0002 for R905A, >0.9999 for H908A, 0.0149 for Q1061A and <0.0001 for R1072A; NBA, 0.0458 for W864A, <0.0001 for R905A, >0.9999 for H908A, 0.0179 for Q1061A, and <0.0001 for R1072A. Averaged raw data are shown in Extended Data Fig. 2b. **e**, Structural comparison of the Ca^2+^–NBA-bound state (purple, PDB 9Z26) and the NC1-bound state (orange, PDB 9Z21) of TRPM4, superimposed using S1–S4 (residues 760–910). NBA and NC1 are shown in stick representation with a transparent envelope, and Ca_TMD_ as a green sphere. The filled orange and blue ellipses mark the top and bottom pockets in the S1–S4 domain, respectively. Arg1072 interacts with both ligands, as indicated by the dashed lines. **f**, Structural comparison of the Ca^2+^–CBA-bound state (blue, PDB 9Z23) and the Ca^2+^-bound warm state (gray, PDB 9B8W) of TRPM4, superimposed using S1–S4 (residues 760–910). CBA and the side chains of H908 and W864 are shown in stick representation, forming a triple stacking interaction indicated by the transparent discs and the red dashed line. Source data

**Extended Data Fig. 8:**
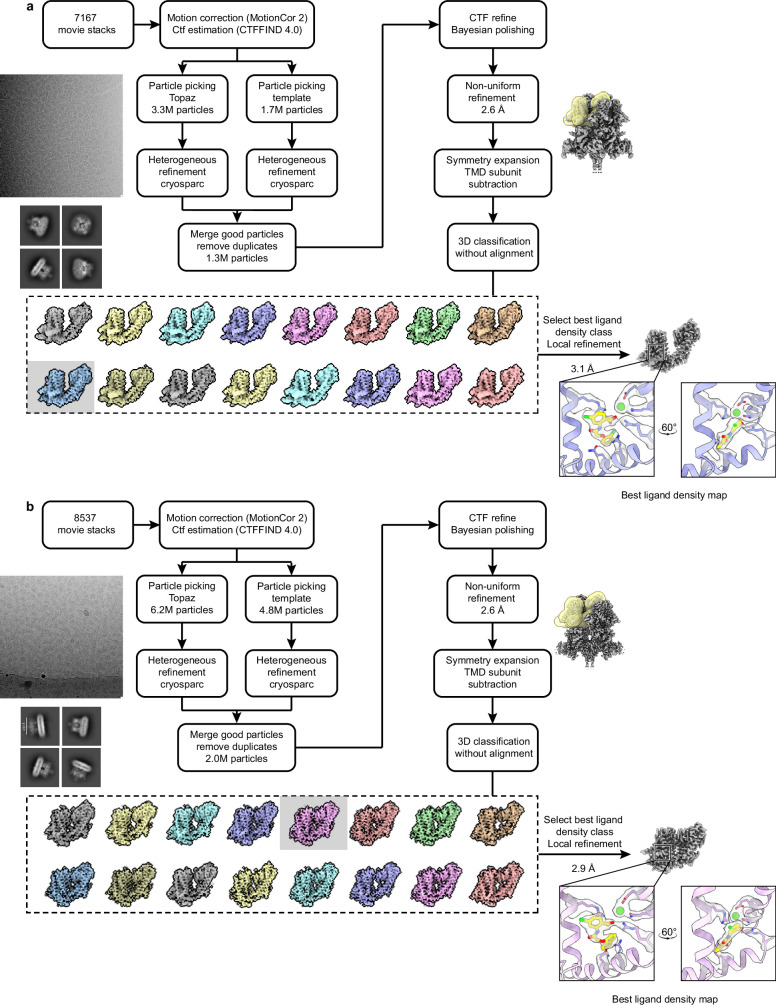
Cryo-EM data processing workflow for the Ca^2+^/NBA–TRPM4–37 °C (a) and Ca^2+^/CBA–TRPM4–37 °C (b) datasets. A representative micrograph for Ca^2+^/NBA–TRPM4–37 °C (**a**, out of 7167 micrographs) and Ca^2+^/CBA–TRPM4–37 °C (**b**, out of 8537 micrographs), and representative 2D classification images are shown. The final consensus map and the TMD-focused map with the best-resolved ligand density are displayed with their respective resolutions.

**Extended Data Fig. 9:**
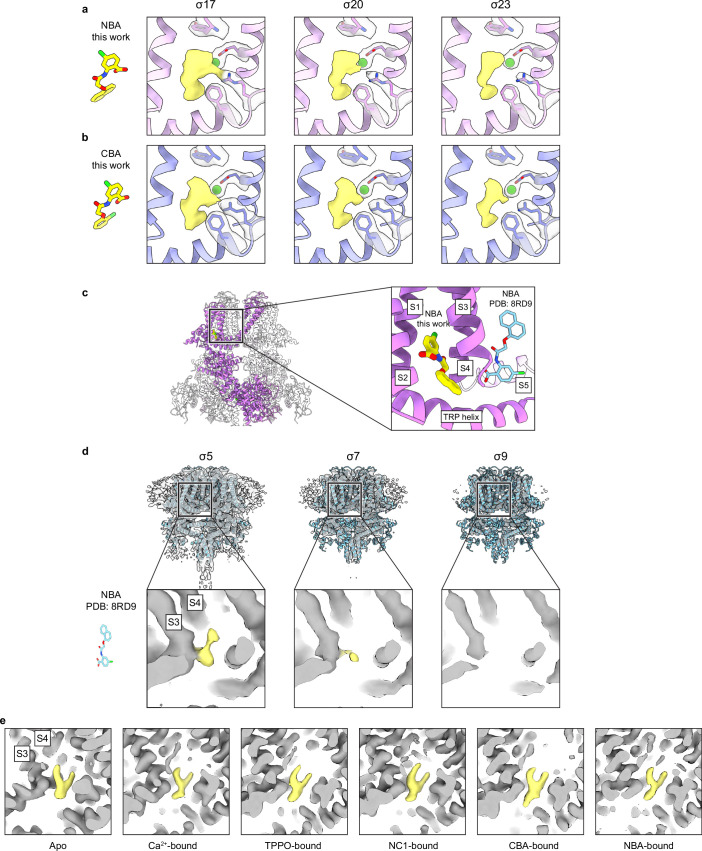
Comparison of the NBA and CBA sites identified in this study with the previously reported NBA site, along with the densities observed within these sites. **a,b**, NBA and CBA densities contoured at different σ levels for the Ca^2+^/NBA–TRPM4–37 °C TMD map and the Ca^2+^/CBA–TRPM4–37 °C TMD map in this study. Densities of NBA and CBA are shown as yellow surfaces. For comparison, densities for Ca_TMD_ and surrounding interacting residues are displayed as transparent surfaces. The NBA and CBA molecules in the poses fitted to the maps are shown on the left in stick representation. **c**, Location of the NBA binding site identified in this work (yellow; PDB-ID 9Z24) compared with the previously reported site (light blue; PDB-ID 8RD9). **d**, The overall cryo-EM map (top) and the densities of the putative NBA binding site (bottom) from the previously published TRPM4 structure in the presence of NBA (EMD-19069), contoured at different σ levels. The putative NBA densities are highlighted in yellow, and the protein densities are shown in gray. The NBA molecule in the pose fitted to the map is shown on the left in stick representation. **e**, Densities at the previously reported NBA binding site in our high-resolution cryo-EM maps across different ligand conditions consistently reveal a Y-shaped density characteristic of a lipid molecule. The Y-shaped densities are highlighted in yellow, and the protein densities are shown in gray.

At 37 °C in the presence of Ca^2+^, cryo-EM analysis of TRPM4 in complex with NBA or CBA uncovered a previously unknown ligand-binding site in the S1–S4 domain in both datasets, present in both cold and warm conformations (Extended Data Fig. 8 and Supplementary Fig. 1). This site lies below the Ca_TMD_ site and adjacent to the S1–S4_upper_ pocket that accommodates TPPO and NC1, and is therefore termed S1–S4_lower_ site (Fig. 5a,b). The ligand densities were of excellent quality (local resolution, ~2.6 Å) and closely matched the crystal structures of NBA and CBA^40^, respectively, allowing unambiguous ligand placement (Supplementary Fig. 1, Extended Data Fig. 9a,b and Supplementary Tables 2 and 3). In both inhibitors, the chlorophenyl-carboxyl group points upward in the S1–S4 domain, with its carbonyl stabilized by two positively charged residues, R905 on S4 and R1072 on the TRP helix (Fig. 5a,b).

Surprisingly, this S1–S4_lower_ site differs from the previously reported NBA-binding site based on lower-resolution cryo-EM maps^41^, which placed the inhibitor ~10 Å away on the opposite face of the S4 helix (Extended Data Fig. 9c)—in the cleft between the S1–S4 domain and the pore domain (Extended Data Fig. 9d), analogous to the vanilloid pocket in TRPV channels and the NDNA site in TRPM5 (refs. ^42,43^). Careful inspection of both regions in our high-resolution maps revealed no evidence supporting the earlier assignment. Instead, our analyses of the NBA- and CBA-bound structures consistently showed a Y-shaped density in the vanilloid pocket, akin to that observed in the apo, TPPO- and NC1-bound structures, indicating a tightly bound endogenous lipid rather than inhibitor occupancy (Extended Data Fig. 9e).

To validate the S1–S4_lower_ pocket functionally, we introduced substitutions at key interacting residues and examined their effects using patch–clamp recordings. Substitution of either R905 or R1072 by alanine completely abolished inhibition (Fig. 5c,d). Moreover, the second aromatic group, a chlorophenyl in CBA or the bulkier naphthyl in NBA, extends downward into a cavity formed between the S4–S5 linker and the TRP helix (Fig. 5a,b). Substitution of Q1061, positioned near this distal aromatic moiety, also markedly reduced inhibition (Fig. 5c,d).

Together, these results establish the S1–S4_lower_ pocket as the functional binding site for NBA and CBA, defining a second pharmacologically active locus in the S1–S4 domain. This domain thus emerges as a bifunctional pharmacological hub in TRPM4, accommodating both activators (TPPO, NC1 and Ca^2+^) and inhibitors (NBA and CBA) through spatially segregated upper and lower sites that converge on shared gating residues, such as R1072, on the TRP helix (Fig. 5e). Consistent with this model, substitution of N786—a residue located within the S1–S4_upper_ region—abolished NC1 activation while leaving NBA-mediated inhibition largely intact (Extended Data Fig. 2c,d). This uncoupling provides functional evidence that the upper and lower S1–S4 pockets are partially independent, despite their close spatial proximity, and can differentially engage ligands to produce distinct pharmacological outcomes.

### NBA and CBA inhibit TRPM4 by locking a pre-open conformation

To elucidate how NBA and CBA inhibit Ca^2+^-dependent activation, we first examined the conformational transitions that underlie canonical TRPM4 gating. In the Ca^2+^-free cold state, W864 on S3 and H908 on S4 form a stabilizing π–π stacking interaction^44–46^. Upon Ca^2+^ binding, coordinated engagement of the Ca_TMD_ and Ca_ICD_ triggers rearrangements in the S1–S4 bundle and TRP helix, repositioning W864 and H908 and thereby disrupting the contact between the intracellular tips of the S3 and S4 helices (Fig. 5f, the gray structure)—a hallmark of Ca^2+^-gated TRPM channel activation^43,47^. This frees the S4–S5 linker, allowing the S5–S6 pore domain to open^10^.

In the Ca^2+^–NBA- and Ca^2+^–CBA-bound structures, this transition is blocked. The distal aromatic moiety of NBA or CBA occupies the position normally adopted by H908 in the activated state, thereby forcing H908 to remain in its apo-like conformation. As a result, H908, W864 and the ligand form a π–π–π triple stacking interaction that restrains the S4–S5 linker and prevents pore opening (Fig. 5f, the purple structure). The overall architecture of the inhibited state closely matches the Ca^2+^-bound warm closed conformation (backbone root mean square deviation, 0.7 Å), indicating that NBA and CBA trap TRPM4 in a Ca^2+^-primed but non-conductive pre-open state.

**Extended Data Fig. 10:**
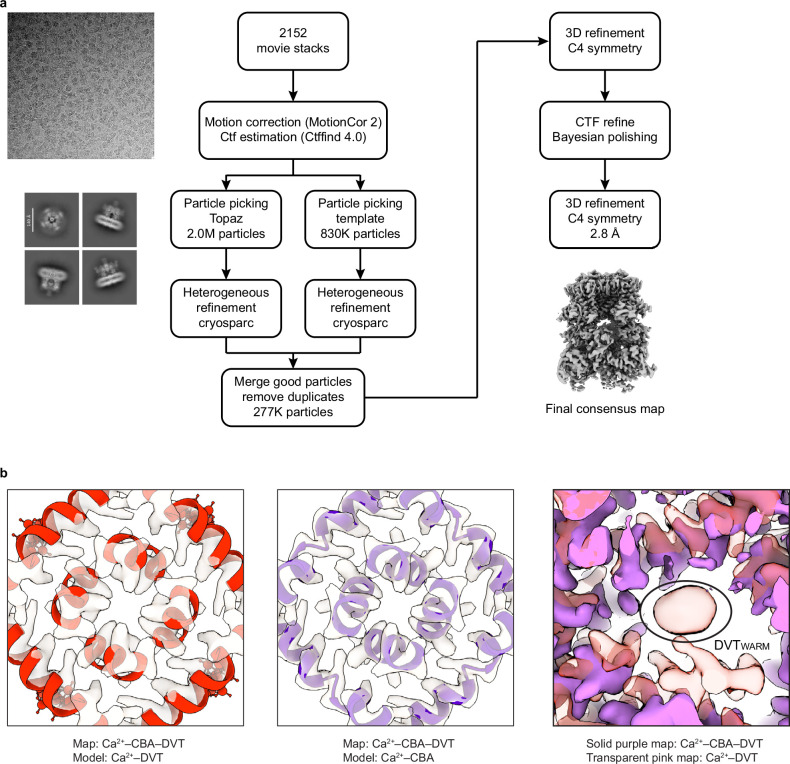
Cryo-EM data processing workflow for the Ca^2+^/DVT/CBA–TRPM4–37 °C dataset. **a**, A representative micrograph (out of 2152 micrographs) and representative 2D classification images are shown. The final map is displayed with the resolution. **b**, Left: superimposition of the Ca^2+^/DVT/CBA–TRPM4 map and the Ca^2+^/DVT–TRPM4_Warm_ model (red; open state), focusing on the transmembrane domain, viewed from the intracellular side. Middle: Superimposition of the Ca^2+^/DVT/CBA–TRPM4 map and the Ca^2+^/CBA–TRPM4 model (purple), focusing on the transmembrane domain, viewed from the intracellular side. Right: superimposition of the cryo-EM maps of Ca^2+^/DVT–TRPM4_Warm_ (transparent pink) and Ca^2+^/DVT/CBA–TRPM4 (solid purple), focusing on the DVT_Warm_ site and its surroundings.

To test this model, we used decavanadate (DVT), a positive modulator that we previously showed binds at the ICD–TMD interface and facilitates the transition of TRPM4 from a Ca^2+^-bound preopen state to the open state by pulling the pore-lining S6 helix to open the ion-conducting pore^10,48^. Notably, DVT binding—and the subsequent transition from the pre-open to the open state—is accompanied by an upward tilting of the intracellular domain toward the transmembrane domain^10^. We therefore reasoned that if NBA and CBA stabilize a Ca^2+^-bound preopen state, they should prevent the conformational changes required for DVT binding and activation, or even preclude DVT binding altogether. To test this prediction, we determined the structure of TRPM4 in the presence of Ca^2+^, DVT and CBA at 37 °C (Supplementary Fig. 1, Extended Data Fig. 10a and Supplementary Table 3). The resulting map contained densities only for Ca^2+^ and CBA, with no evidence of DVT binding, and the protein conformation was identical to the Ca^2+^–CBA-bound closed state (Extended Data Fig. 10b). These data provide direct structural evidence that DVT cannot override NBA or CBA inhibition, supporting our proposed inhibitory mechanism.

Interestingly, this mechanism converges with that of ATP, which also prevents opening by stabilizing the pre-open state, albeit through a distinct intracellular binding site^10^. Unlike ATP, however, NBA and CBA act from the extracellular side and maintain high potency at physiological temperature, offering both accessibility and stability in the native conformational landscape. Thus, NBA and CBA represent promising scaffolds for the design of TRPM4 inhibitors with therapeutic potential.

## Discussion

This study reveals that TRPM4 pharmacology is not static but dynamically shaped by the physiological environment, with temperature and intracellular Ca^2+^ acting as active determinants of ligand binding, efficacy and mechanism. By integrating single-particle cryo-EM and electrophysiology, we reveal that these intrinsic factors cooperatively remodel the conformational landscape of TRPM4, exposing latent binding sites and revealing context-dependent pharmacological behaviors that are obscured under non-physiological conditions.

**Fig. 6:**
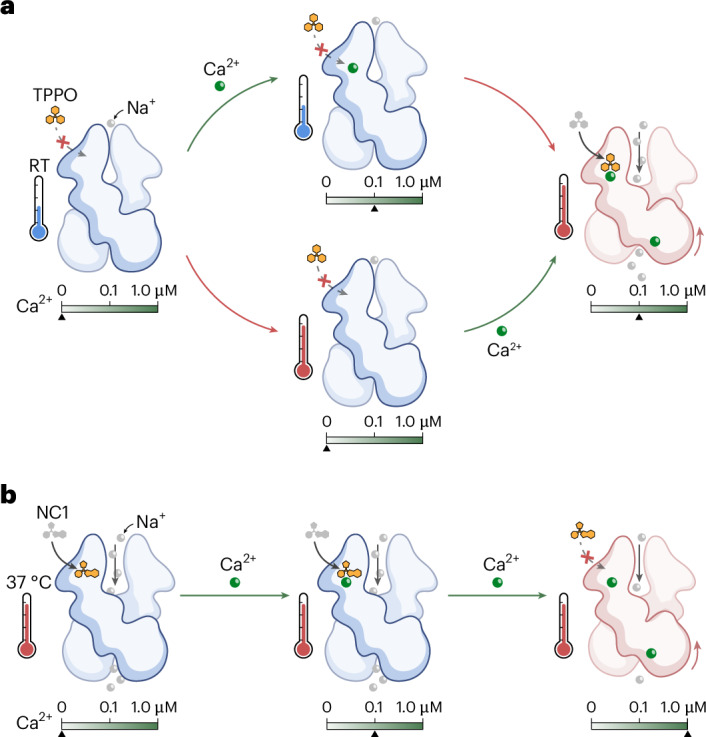
Distinct temperature- and Ca^2+^-dependent activation mechanisms of TPPO and NC1 on TRPM4. **a**, Schematic of the polymodal activation of TRPM4 by TPPO, Ca^2+^ and temperature. Neither resting Ca^2+^ levels nor physiological temperature alone is sufficient to enable TPPO-mediated activation. Only when both resting Ca^2+^ and physiological temperature are present can TPPO exert its activating effect. **b**, Schematic of NC1 activity, which functions independently of Ca^2+^ at resting levels but loses efficacy once intracellular Ca^2+^ rises to stimulatory concentrations.

The physiological context unmasks the true pharmacological profiles of ligands. The small molecule TPPO, long regarded as a TRPM5-specific inhibitor, emerges here as a potent activator of TRPM4, but only when both temperature and intracellular Ca^2+^ reach physiological levels (Fig. 6a). Conversely, NC1, previously characterized as a Ca^2+^-independent agonist, undergoes a functional reversal: at basal Ca^2+^ concentrations, it activates TRPM4, whereas at elevated Ca^2+^, it becomes functionally antagonized, contributing little to no additional effect beyond Ca^2+^ alone, revealing an unexpected inhibitory facet of Ca^2+^ regulation (Fig. 6b). Mechanistically, these contrasting behaviors arise because temperature and Ca^2+^ cooperatively determine the equilibrium between cold and warm conformations of TRPM4, thereby governing access to key residues in the S1–S4 regulatory domain. Thus, ligand efficacy in TRPM4 is not an intrinsic property of the compound alone, but an emergent consequence of its interplay with the conformational and thermodynamic landscape of the channel.

Moreover, our structural analyses establish the S1–S4 domain as a versatile pharmacological hub that integrates both environmental and chemical inputs. In this domain, around the Ca_TMD_ site, two spatially distinct but functionally coupled sites control opposite gating outcomes: the S1–S4_upper_ pocket, occupied by activators TPPO, NC1 and deacetyl bisacodyl^25^, and the S1–S4_lower_ pocket, targeted by inhibitors NBA and CBA (Fig. 5e). Both pockets converge on the TRP helix, with the conserved residue R1072 acting as a central allosteric node that links environmental cues to gating transitions. Ligand engagement at the upper pocket promotes activation by coupling between the S1–S4 domain and the pore domain, whereas ligand binding to the lower pocket disrupts this coupling, stabilizing a non-conductive state. This dual-site organization provides a unified explanation for how temperature, Ca^2+^ and chemically diverse ligands—activators and inhibitors alike—modulate TRPM4 through a shared structural framework. Of note, our TPPO- and NC1-bound cryo-EM structures capture TRPM4 in a closed pore conformation, consistent with our previous observations that the pore remains closed in the presence of the endogenous activator Ca^2+^ alone. These observations suggest that agonist engagement with the S1–S4 domain, although necessary for activation, is not sufficient to stabilize an open pore under cryo-EM conditions, likely owing to the absence of membrane potential. By contrast, open-pore conformations have been observed in the presence of additional modulators, such as DVT or PIP_2_, which render the channel voltage independent^10,49^.

This regulatory logic uncovered here could represent a general feature of TRPM channels. In TRPM3 and TRPM5, both agonists and antagonists occupy the upper pocket^50–52^, and in TRPM8, ligands engage both^53–55^. The S1–S4 domain thus exemplifies an evolutionary design in which temperature and chemical cues dynamically reconfigure a common structural scaffold to yield distinct, context-dependent functional outcomes.

Notably, we found that temperature-dependent ligand recognition in TRPM4 is not limited to regions that undergo large-scale rearrangements. Even sites in structurally stable domains—such as the S1–S4 transmembrane bundle, which exhibits only subtle temperature-driven movements—can undergo marked shifts in ligand accessibility and efficacy. This principle likely extends to many drug targets, highlighting that seemingly rigid structural regions could harbor cryptic, environment-sensitive pharmacology.

More broadly, these findings support an environment-aware framework of pharmacology, in which physiological variables, such as temperature and intracellular Ca^2+^ levels, act as active modulators of ligand binding, potency and efficacy rather than as passive background conditions. At present, however, the extent to which this principle applies across proteins more generally remains underexplored. Modulation of ligand binding by temperature—and potentially by Ca^2+^ or other physiological cofactors—has historically received limited attention, and relatively few explicit examples are available.

Nevertheless, precedent does exist. Crystallographic studies of PBP1B have shown that ligands adopt distinct binding poses at cryogenic and room temperatures^56^, and analogous temperature-dependent side-chain rearrangements upon metal binding have been observed in hen egg-white lysozyme^57^. Similarly, a recent study of ionotropic glutamate receptors has shown that glutamate-dependent pore opening is strongly promoted at physiological temperatures, allowing activated states to be captured by cryo-EM that were not observed in samples prepared under non-physiological temperature conditions^9^. Together with these examples, our results suggest that pharmacological specificity could emerge from the interplay between ligand chemistry and the dynamic, context-dependent structural state of the target protein. We propose that extending similar approaches to other ion channels and proteins will be an important direction for future work, with potential implications for bridging the gap between in vitro pharmacological screening and in vivo drug performance.

For ubiquitously expressed proteins such as TRPM4, these principles carry important therapeutic implications. By exploiting local physiological changes—such as elevated intracellular Ca^2+^ levels during stress or altered thermal states in disease—future drugs could be tailored to act selectively in pathological contexts while sparing normal function. Such environment-sensitive design strategies could reduce side effects and offer a new route toward precision pharmacology.

## Methods

### Human TRPM4 protein expression and purification

The gene encoding human full-length TRPM4 (UniProtKB: Q8TD43) was subcloned into pEG BacMam vector with a 2×Strep tag, GFP and a thrombin-cleavage site at the amino terminus, as previous described^10,58^. Substitutions in TRPM4 were generated using primers described in Supplementary Table 4. Bacmid and baculovirus of TRPM4 in a BacMam vector were generated, and P2 viruses were used to infect tsA cells (purchased from the American Type Culture Collection (cat. no. CRL-3216) and were not authenticated experimentally in this study) grown in Freestyle 293 expression medium (Gibco) in suspension culture. Cells were incubated at 37 °C for 8 h, 10 mM sodium butyrate was added to the culture and the temperature was lowered to 30 °C. The cells were collected 72 h after infection and resuspended in a buffer containing 100 mM Tris pH 8.0 and 150 mM NaCl (TBS buffer) in the presence of 1 mM phenylmethylsulphonyl fluoride, 0.8 μM aprotinin, 2 μg ml^−1^ leupeptin and 2 mM pepstatin A. The cells were lysed by sonication and the membrane fraction was collected by centrifugation at 186,000*g* using a 45 Ti rotor (Beckman Coulter) for 1 h at 4 °C. The membrane was then homogenized with a Dounce homogenizer in TBS buffer supplemented with protease inhibitors. The protein was extracted from the membrane with TBS buffer supplemented with 1% GDN and protease inhibitors for 3 h at 4 °C. The solubilized proteins were loaded to Strep-Tactin resin. After being washed with TBS buffer supplemented with 0.02% GDN, TRPM4 was eluted with the same buffer, supplemented with 10 mM desthiobiotin. The GFP tag was cleaved, and proteins were concentrated and further purified by size-exclusion chromatography (Superose 6 Increase 10/300 GL). The peak fractions containing the TRPM4 were pooled and concentrated to 8 mg ml^−1^.

### EM sample preparation and data acquisition

Purified TRPM4 was incubated with 5 mM calcium chloride or 5 mM EGTA, as required for each experiment, for 30 s at 18 or 37 °C. Then, 0.1 mM TPPO was added and incubated for a further 2 min at 18 or 37 °C; alternatively, 0.25 mM CBA or NBA, or 0.8 mM NC1, was added and incubated for a further 2 min at 37 °C. A 2.5-µl sample was applied to a glow-discharged Quantifoil holey carbon grid (gold, 2/1 μM size/hole space, 300 mesh). The grids were blotted for 1.5 s in the Vitrobot Mark III set to 100% humidity at 18 °C or 37 °C, with a 15 s wait time before being plunge-frozen into liquid ethane cooled by liquid nitrogen.

For all samples, images were recorded using the FEI Glacios electron microscope at 200 kV and a nominal magnification of ×135,000. A Falcon 4i direct electron detector with Selectris energy filter was used resulting a pixel size of 0.87 Å or 0.874 Å. EPU was used for automated acquisition. Nominal defocus ranged from −0.5 to −1.4 μM.

### Cryo-electron microscopy data analysis procedure

The detailed workflow for the data-processing procedure is summarized in Extended Data Figs. 4, 5, 7, 8 and 10. In general, the raw movies for each dataset were motion-corrected using MotionCor2 (v.1.1.0)^59^. The per-micrograph defocus values were estimated using ctffind (v.4.1.10)^60^. Particle picking was performed using relion template picker^61^ and topaz (v.0.2.4)^62^. Junk particles were removed by rounds of 3D heterogeneous refinement using CryoSPARC^63^. Good particles were selected for non-uniformed refinement with *C*_4_ symmetry in CryoSPARC to generate a 3D map. Multiple rounds of CTF refinement and Bayesian polishing were performed in RELION and CryoSPARC to further improve the map resolution.

For the Ca^2+^–TPPO (37 °C) dataset, we performed symmetry expansion at the single-subunit level of the ICD from a map refined with *C*_4_ symmetry, followed by monomer ICD subtraction. The subtracted images of the monomer ICD were subjected to 3D classification without image alignment in RELION to identify the warm and cold conformations. The corresponding warm and cold monomers subsequently underwent similar TMD monomer 3D classification to identify the map with the bound ligand with the best local resolution.

For the Ca^2+^–CBA (37 °C) and Ca^2+^–NBA (37 °C) datasets, symmetry expansion at the single-subunit level of the TMD was done from the map refined with *C*_4_ symmetry, followed by monomer TMD subtraction. The subtracted images of the monomer TMD were subjected to 3D classification in RELION, without image alignment, to identify the class exhibiting the map with bound ligand with the best local resolution.

Map resolution estimates were based on the gold standard Fourier shell correlation 0.143 criterion for all datasets.

### Model building

Atomic models were generated by rigid-body fitting of the TMD, MHR1, MHR2, MHR3 and MHR4 and carboxy-terminal domains from a published human TRPM4 model (PDB 5WP6) into the final cryo-EM maps. Ligands were fitted into the density through real-space refinement using COOT^64^. The CIF file of ligands was generated using grade web server (Global Phasing). The models were then manually adjusted in COOT and subjected to phenix.real_space_refine^65^ to improve the model metrics. The final models were validated using phenix.molprobity^66^. Figures were generated using UCSF ChimeraX^67^.

### Electrophysiology

TsA201 cells expressing plasmids encoding N-terminal GFP-tagged human TRPM4 WT and mutants were used. One day after transfection with plasmid DNA (100 ng ml^−1^) and Lipofectamine 2000 (Invitrogen, cat. no. 11668019), the cells were trypsinized and replated onto poly-L-lysine-coated (Sigma, cat. no. P4707) glass coverslips. After cell attachment, the coverslip was transferred to a recording chamber. Whole-cell patch–clamp recordings were performed at room temperature (21–23 °C) or body temperature (36–38 °C). The temperature of perfusion solutions was controlled by thermal control devices (cat. no. SC-20/CL-100, Warner Instruments).

For whole-cell recordings, glass pipettes were pulled to 3–5 MΩ and filled with an internal solution. Signals were amplified with a MultiClamp 700B amplifier (controlled by MultiClamp Commander v2.2.2.2) and digitized using a Digidata 1550B A/D converter controlled by Clampex v11.3 (Molecular Devices). Whole-cell currents were measured in cells with an access resistance of <10 MΩ after achieving whole-cell configuration. Whole-cell capacitance was compensated by the amplifier circuitry. Whole-cell currents were recorded using a two-step voltage protocol in which cells were held at –100 mV for 200 ms and then stepped to +100 mV for another 200 ms, and this was repeated every 10 s. An alternative voltage-step protocol was applied, in which cells were stepped from –100 mV to +140 mV in 20-mV increments, with each step lasting 100 ms. A voltage ramp from –100 to +100 mV (200 ms duration) was applied every 5 s from a holding potential of 0 mV. Data were sampled at 10 kHz and low-pass filtered at 2 kHz. Recordings were analyzed using Clampfit v11.3 (Axon Instruments), GraphPad Prism v10.6.1 (GraphPad Software) and OriginPro 2024 (OriginLab). Data were also analyzed using custom Python scripts, including pyABF for loading and processing ABF electrophysiology files. Electrophysiology files were parsed and analyzed in Python v3.11.8 executed in Spyder v6.0.7 (Anaconda environment) on macOS v26.0.1 (ARM architecture). For data processing, we used pandas v2.2.2, and we used NumPy v1.26.4 for numerical operations. All custom analysis scripts used in this study are available at https://github.com/junuk861/ephys-analysis-scripts (version v1.0.1) and have been archived on Zenodo (10.5281/zenodo.19711152)^68^. The bath solution (pH 7.4) contained 150 mM NaCl, 3 mM KCl, 10 mM HEPES, 2 mM CaCl_2_, 1 mM MgCl_2_ and 12 mM mannitol. Patch pipettes were filled with internal solutions containing 150 mM NaCl, 1 mM MgCl_2_, 10 mM HEPES, 5 mM EGTA and CaCl_2_ adjusted (0 or 1.69 mM) to yield 0 or 0.1 µM free Ca^2+^, respectively (pH 7.3, 22 °C) (source: https://somapp.ucdmc.ucdavis.edu/pharmacology/bers/maxchelator/CaEGTA-TS.htm). For whole-cell recordings of NC1-induced and NBA- or CBA-inhibited currents, the extracellular solution contained the following (in mM): 150 NaCl, 10 HEPES, 2 CaCl_2_ and 1 MgCl_2_. The intracellular solution contained (in mM): 150 NaCl, 10 HEPES and 5 EGTA. The intracellular concentration of CaCl_2_ was set to either 0, 1.69 or 4.45 mM (corresponding to 0, 0.1 or 1 µM of free Ca^2+^). The pH was adjusted to 7.4 using NaOH in both the extracellular and intracellular solutions.

TPPO (Sigma-Aldrich, cat. no. T84603) was prepared daily as a 70 mM stock solution in ethanol and diluted to the desired final concentration in the bath solution immediately before use. NC1 (Necrocide 1, MCE, HY-14307), NBA (4-chloro-2-(2-(naphthalen-1-yloxy)acetamido)benzoic acid, MCE, HY-128172) and CBA (4-chloro-2-[2-(2-chloro-phenoxy)-acetylamino]-benzoic acid, Tocris, 6724) were dissolved in DMSO to a stock concentration of 10, 100, and 50 mM, respectively.

### Surface protein labeling

Surface protein labeling experiments were performed using the Pierce Cell Surface Protein Isolation Kit (Thermo Fisher Scientific). Surface and total protein levels of wild-type and mutant TRPM4 were assessed by in-gel fluorescence of the N-terminal GFP tag.

### Statistics and reproducibility

For electrophysiology, each recording was obtained from a single cell using the whole-cell patch–clamp technique, and the recording unit (*n*) represents the number of cells. Details on experimental design, including whether comparisons were performed between cells or in the same cell and the use of independent cells for each condition, are described in the figure legends and main text. Experiments were repeated across multiple days using independent cell preparations to account for batch-to-batch variability. Statistical analyses were performed using standard methods, as described in the relevant method section and figure legends. Data are presented as mean ± s.e.m. unless otherwise indicated. The number of biologically independent experimental replicates or measurements are indicated in the figure legend.

For cryo-EM, single-particle analysis was used for structure determination. Purified samples were vitrified using standard plunge-freezing methods, and automated data collection and data processing followed established protocols in the field, and detailed descriptions are in methods and extended data figures. Micrograph and particle counts are listed in Supplementary Tables 1–3. Statistical analyses were performed using standard methods, as described in the relevant figure legends. All attempts to replicate the structural findings were successful.

No statistical method was used to predetermine sample size. Sample sizes were similar to those generally used in the field for electrophysiological recordings and cryo-EM imaging. No data were excluded from the electrophysiology analyses. During cryo-EM data processing, particles that clearly did not show target-protein-like features or showed broken or disordered protein domain(s) were excluded, consistent with common practice. The experiments were not randomized. The investigators were not blinded to allocation during experiments and outcome assessment.

### Reporting summary

Further information on research design is available in the Nature Portfolio Reporting Summary linked to this article.

## Online content

Any methods, additional references, Nature Portfolio reporting summaries, source data, extended data, supplementary information, acknowledgements, peer review information; details of author contributions and competing interests; and statements of data and code availability are available at 10.1038/s41594-026-01818-3.

## Supplementary information

Supplementary Information Supplementary Fig. 1 and Tables 1–4.

Reporting Summary

Peer Review File

## Source data

Source Data Fig. 1 Electrophysiology source data for Fig. 1a–i.

Source Data Fig. 2 Electrophysiology source data for Fig. 2a–i.

Source Data Fig. 4 Electrophysiology source data for Fig. 4c.

Source Data Fig. 4 Uncropped gel in Fig. 4c.

Source Data Fig. 5 Electrophysiology source data for Fig. 5c,d.

Source Data Extended Data Fig. 1 Electrophysiology source data for Extended Data Fig. 1a–k.

Source Data Extended Data Fig. 1 Uncropped gel in Extended Data Fig. 1j.

Source Data Extended Data Fig. 2 Electrophysiology source data for Extended Data Fig. 2a,b,d.

Source Data Extended Data Fig. 3 Electrophysiology source data for Extended Data Fig. 3a–c.

## Data Availability

Cryo-EM density maps have been deposited at the EMDB (Electron Microscopy Data Bank) and the Research Collaboratory for Structural Bioinformatics Protein Data Bank (RCS-PDB), respectively. EMDB and PDB accession codes are as follows: Ca^2+^–TPPO–TRPM4 (37 °C) is EMDB-73754, PDB 9Z1W; Ca^2+^–TPPO–TRPM4 (37 °C) warm TMD is EMDB-73755, PDB 9Z1X; Ca^2+^–TPPO–TRPM4 (37 °C) cold TMD is EMDB-73756, PDB 9Z1Y; Ca^2+^–TPPO–TRPM4 (18 °C) is EMDB-73757, PDB 9Z1Z; EGTA–TPPO–TRPM4 (37 °C) is EMDB-73758, PDB 9Z20; Ca^2+^–NC1–TRPM4 (37 °C) is EMDB-73759, PDB 9Z21; EGTA–NC1–TRPM4 (37 °C) is EMDB-73760, PDB 9Z22; Ca^2+^–CBA–TRPM4 (37 °C) is EMDB-73761, PDB 9Z23; Ca^2+^–CBA–TRPM4 (37 °C) TMD is EMDB-73762, PDB 9Z24; Ca^2+^–NBA–TRPM4 (37 °C) is EMDB-73763, PDB 9Z25; Ca^2+^–NBA–TRPM4 (37 °C) TMD is EMDB-73764, PDB 9Z26; Ca^2+^–CBA–DVT–TRPM4 (37 °C) is EMDB-73765, PDB 9Z27. Data and materials can be obtained from the corresponding authors upon request. Source data are provided with this paper.

## Code availability

All custom analysis scripts used in this study are available at https://github.com/junuk861/ephys-analysis-scripts (version v1.0.1) and have been archived on Zenodo (10.5281/zenodo.19711152)^68^.
