# Supplementary material for: Temperature and intrinsic Ca2+ reshape TRPM4 pharmacology
Source: Nat Struct Mol Biol. 2026 Jun 9;33(6):973–84. doi: 10.1038/s41594-026-01818-3 (PMC13275316; doi:10.1038/s41594-026-01818-3)
Supplement: Supplementary file 1 — Supplementary Fig. 1 and Tables 1–4. [file 41594_2026_1818_MOESM1_ESM.pdf]

nature structural & molecular biology

Supplementary information https://doi.org/10.1038/s41594-026-01818-3

Temperature and intrinsic Ca^{2+} reshape TRPM4 pharmacology

In the format provided by the authors and unedited

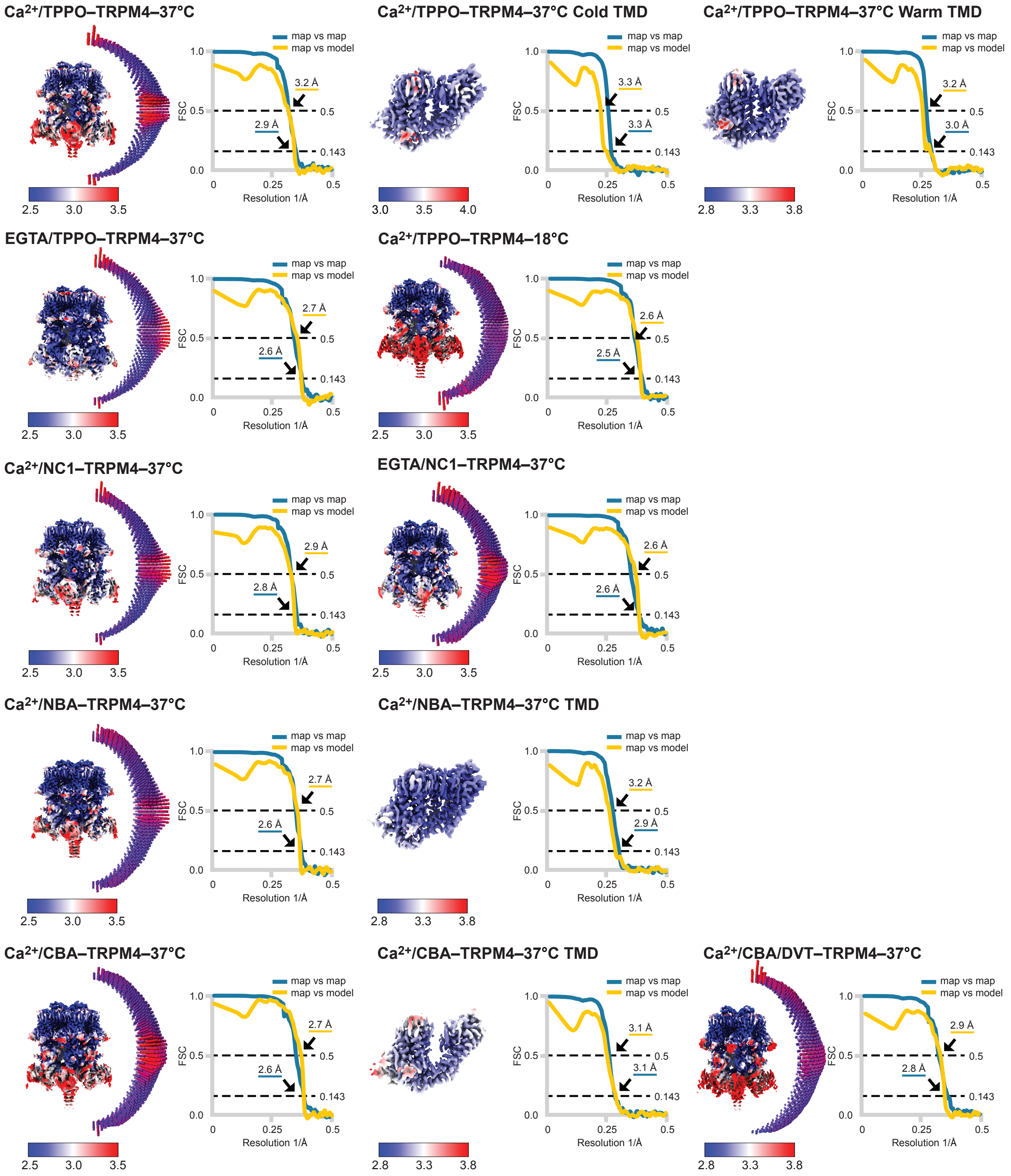

Supplementary Figure 1. Assessment of cryo-EM data quality. For each map, left: cryo-EM maps colored by local resolution; right: Fourier shell correlation (FSC) plot.

Cryo-EM data collection, refinement and validation statistics

	Ca^{2+}/TPPO–TRPM4–37°C (EMDB-73754) (PDB 9Z1W)	Ca^{2+}/TPPO–TRPM4–37°C warm TMD (EMDB-73755) (PDB 9Z1X)	Ca^{2+}/TPPO–TRPM4–37°C cold TMD (EMDB-73756) (PDB 9Z1Y)	Ca^{2+}/TPPO–TRPM4–18°C (EMDB-73757) (PDB 9Z1Z)	EGTA/TPPO–TRPM4–37°C (EMDB-73758) (PDB 9Z20)
**Data collection and processing**					
Magnification	135000	135000	135000	135000	135000
Voltage (kV)	200	200	200	200	200
Electron exposure (e–/Å^2)	40	40	40	40	40
Defocus range (µm)	-0.5 – -1.4	-0.5 – -1.4	-0.5 – -1.4	-0.5 – -1.4	-0.5 – -1.4
Pixel size (Å)	0.87	0.87	0.87	0.87	0.874
Symmetry imposed	C4	C1	C1	C1	C4
Initial particle images (no.)	2.6M			2.7M	4.9M
Final particle images (no.)	1180K	882K	294K	760K	1929K
Map resolution (Å)	2.9	3.0	3.3	2.6	2.5
FSC threshold	0.143	0.143	0.143	0.143	0.143
Map resolution range (Å)	2.9 – 246.2	3.0 – 246.2	3.2 – 246.2	2.6 – 246.2	2.5 – 246.2
**Refinement**					
Initial model used (PDB code)	5WP6	5WP6	5WP6	5WP6	5WP6
Model resolution (Å)	3.3	3.3	3.5	3.1	2.7
FSC threshold	0.5	0.5	0.5	0.5	0.5
Model resolution range (Å)	3.3 – 246.2	3.3 – 246.2	3.5 – 246.2	3.1 – 246.2	2.7 – 246.2
Map sharpening *B* factor (Å^2)	-120	-30	-30	-100	-100
Model composition					
Non-hydrogen atoms	30220	2912	2912	29272	29312
Protein residues	3976	365	365	3892	3896
Ligands	12	2	2	12	0
B factors (Å^2)					
Protein	133.28	83.39	82.41	123.92	103.06
Ligand	144.31	98.78	95.88	78.23	
R.m.s. deviations					
Bond lengths (Å)	0.004	0.003	0.002	0.006	0.003
Bond angles (°)	0.469	0.448	0.419	0.493	0.452
Validation					
MolProbity score	1.21	1.13	1.10	1.14	0.92
Clashscore	1.89	2.41	2.24	1.69	0.97
Poor rotamers (%)	0.68	0	0	0.5	1.13
Ramachandran plot					
Favored (%)	96.15	97.49	97.49	96.63	97.58
Allowed (%)	3.85	2.51	2.51	3.37	2.42
Disallowed (%)	0	0	0	0	0

Supplementary Table 1: Cryo-EM data collection, refinement and validation statistics. Data are shown for Ca^{2+}/TPPO–TRPM4–37°C, Ca^{2+}/TPPO–TRPM4–37°C warm TMD, Ca^{2+}/TPPO–TRPM4–37°C cold TMD, Ca^{2+}/TPPO–TRPM4–18°C, and EGTA/TPPO–TRPM4–37°C.

	Ca^{2+}/NC1–TRPM4–37°C (EMDB-73759) (PDB 9Z21)	EGTA/NC1–TRPM4–37°C (EMDB-73760) (PDB 9Z22)	Ca^{2+}/CBA–TRPM4–37°C (EMDB-73761) (PDB 9Z23)	Ca^{2+}/CBA–TRPM4–37°C TMD (EMDB-73762) (PDB 9Z24)	Ca^{2+}/NBA–TRPM4–37°C (EMDB-73763) (PDB 9Z25)
**Data collection and processing**					
Magnification	135000	135000	135000	135000	135000
Voltage (kV)	200	200	200	200	200
Electron exposure (e–/Å^2)	40	40	40	40	40
Defocus range (μm)	-0.5 – -1.4	-0.5 – -1.4	-0.5 – -1.4	-0.5 – -1.4	-0.5 – -1.4
Pixel size (Å)	0.874	0.87	0.87	0.87	0.874
Symmetry imposed	C4	C4	C4	C1	C4
Initial particle images (no.)	5.6M	4.2M	3.3M		6.2M
Final particle images (no.)	1636K	1475K	1290K	160K	2049K
Map resolution (Å)	2.8	2.6	2.6	3.1	2.6
FSC threshold	0.143	0.143	0.143	0.143	0.143
Map resolution range (Å)	2.8 – 246.2	2.6 – 246.2	2.6 – 246.2	3.1 – 246.2	2.6 – 246.2
**Refinement**					
Initial model used (PDB code)	5WP6	5WP6	5WP6	5WP6	5WP6
Model resolution (Å)	3.1	2.9	3.2	3.3	2.9
FSC threshold	0.5	0.5	0.5	0.5	0.5
Model resolution range (Å)	3.1 – 246.2	2.9 – 246.2	3.2 – 246.2	3.3 – 246.2	2.9 – 246.2
Map sharpening *B* factor (Å^2)	-117	-105	-102	-50	-106
Model composition					
Non-hydrogen atoms	30300	29396	30304	2740	30300
Protein residues	3976	3900	3972	338	3972
Ligands	12	4	12	2	12
B factors (Å^2)					
Protein	107.36	100.17	108.2	101.63	114.79
Ligand	117.04	83.25	66.02	116.92	82.15
R.m.s. deviations					
Bond lengths (Å)	0.004	0.004	0.004	0.004	0.003
Bond angles (°)	0.488	0.439	0.460	0.468	0.440
Validation					
MolProbity score	1.36	1.44	1.22	1.2	1.34
Clashscore	2.14	4.57	1.6	2.19	2.35
Poor rotamers (%)	0.27	0.14	1.08	0.35	0.27
Ramachandran plot					
Favored (%)	94.62	96.74	95.79	96.69	95.28
Allowed (%)	5.38	3.26	4.21	3.31	4.72
Disallowed (%)	0	0	0	0	0

Supplementary Table 2: Cryo-EM data collection, refinement and validation statistics. Data are shown for Ca^{2+}/NC1–TRPM4–37°C, EGTA/NC1–TRPM4–37°C, Ca^{2+}/CBA–TRPM4–37°C, Ca^{2+}/CBA–TRPM4–37°C TMD, and Ca^{2+}/NBA–TRPM4–37°C.

	Ca^{2+}/NBA–TRPM4–37°C TMD (EMDB-73764) (PDB 9Z26)	Ca^{2+}/CBA/DVT–TRPM4–37°C (EMDB-73765) (PDB 9Z27)
**Data collection and processing**		
Magnification	135000	135000
Voltage (kV)	200	200
Electron exposure (e–/Å^2)	40	40
Defocus range (μm)	-0.5 – -1.4	-0.5 – -1.4
Pixel size (Å)	0.874	0.87
Symmetry imposed	C1	C4
Initial particle images (no.)		2M
Final particle images (no.)	239K	278K
Map resolution (Å)	2.9	2.8
FSC threshold	0.143	0.143
Map resolution range (Å)	2.9 – 246.2	3.8 – 246.2
**Refinement**		
Initial model used (PDB code)	5WP6	5WP6
Model resolution (Å)	3.2	3.2
FSC threshold	0.5	0.5
Model resolution range (Å)	3.2 – 246.2	3.2 – 246.2
Map sharpening *B* factor (Å^2)	-50	-95
Model composition		
Non-hydrogen atoms	2945	30292
Protein residues	364	3972
Ligands	2	12
B factors (Å^2)		
Protein	110.32	191.28
Ligand	125.37	118.86
R.m.s. deviations		
Bond lengths (Å)	0.004	0.002
Bond angles (°)	0.499	0.466
Validation		
MolProbity score	1.11	1.34
Clashscore	2.72	3.37
Poor rotamers (%)	0.97	0.54
Ramachandran plot		
Favored (%)	97.77	96.64
Allowed (%)	2.23	3.36
Disallowed (%)	0	0

Supplementary Table 3: Cryo-EM data collection, refinement and validation statistics. Data are shown for Ca^{2+}/NBA–TRPM4–37°C TMD, and Ca^{2+}/DVT/CBA–TRPM4–37°C.

Oligonucleotide sequence for mutagenesis

Oligonucleotide ID	Oligonucelotide sequence
R1072A-F	gttccactctgcgcccgcattagcacctcccttcattg
R1072A-R	ctaatgcgggcgcagagtggaactctctaattaatc
Y790A-F	cgtagttagcgcgttattgtttttgctgttatttag
Y790A-R	caaaaacaataacgcgctaactacgttacccataaaaattg
W820A-F	gctttacttcgcggccttcaccctcttgtgtgaag
W820A-R	gggtgaaggccgcgaagtaaagcaagagttcaagac
R905A-F	ctttaccgtggcactgttacacatcttcaccgtgaac
R905A-R	gatgtgtaacagtgccacggtaaagaccatgaaatc
F793A-F	ctacttattggctttgctgttatttagtagagttttg
F793A-R	aataacagcaaagccaataagtagctaactacgttacc
F902A-F	gatttcatggtcgctaccgtgagactgttacacatc
F902A-R	gtctcacggtagcgaccatgaaatctatgcataac
D868A-F	gaaccagtgtgccttagtagccttgacctgcttctt
D868A-R	ggctactaaggcacactggttccagctatcagctaag
F1069A-F	taattagagaggcccactctaggcccgcattagcac
F1069A-R	gcctagagtgggcctctctaattaatctgtaccgctg
L824A-F	ggccttcaccgccttgtgtgaagagcttagacaggg
L824A-R	cttcacacaaggcggtgaaggcccagaagtaaagc
W864A-F	gctgatagcgcgaaccagtgtgacttagtagccttg
W864A-R	cacactggttcgcgctatcagctaagtataatcttaac
H908A-F	gagactgttagccatcttcaccgtgaacaagcagttag
H908A-R	cggtgaagatggctaacagtctcacggtaaagacc
Q1061A-F	ctggaaggcagcgcggtacagattaattagagagttc
Q1061A-R	aatctgtaccgcgctgccttccagtaaaggtcagag
N786A-F	ttttatgggtgcggtagttagctacttattgtttttg
N786A-R	gctaactaccgcacccataaaaattgtcactgg
S789A-F	gtaacgtagttgcctacttattgtttttgctgttatt
S789A-R	caataagtaggcaactacgttacccataaaaattg

Supplementary Table 4: Oligonucleotide sequence for generate TRPM4 mutations
