# Supplementary material for: Temperature and intrinsic Ca2+ reshape TRPM4 pharmacology
Source: Nat Struct Mol Biol. 2026 Jun 9;33(6):973–84. doi: 10.1038/s41594-026-01818-3 (PMC13275316; doi:10.1038/s41594-026-01818-3)
Supplement: Supplementary file 2 — Reporting Summary [file 41594_2026_1818_MOESM2_ESM.pdf]

Corresponding author(s): Juan Du & Wei Lü

Last updated by author(s): Apr 24, 2026

Reporting Summary

Nature Portfolio wishes to improve the reproducibility of the work that we publish. This form provides structure for consistency and transparency in reporting. For further information on Nature Portfolio policies, see our Editorial Policies and the Editorial Policy Checklist.

Statistics

For all statistical analyses, confirm that the following items are present in the figure legend, table legend, main text, or Methods section.

n/a	Confirmed	
☐	☒	The exact sample size (*n*) for each experimental group/condition, given as a discrete number and unit of measurement
☐	☒	A statement on whether measurements were taken from distinct samples or whether the same sample was measured repeatedly
☐	☒	The statistical test(s) used AND whether they are one- or two-sided<br>*Only common tests should be described solely by name; describe more complex techniques in the Methods section.*
☒	☐	A description of all covariates tested
☒	☐	A description of any assumptions or corrections, such as tests of normality and adjustment for multiple comparisons
☐	☒	A full description of the statistical parameters including central tendency (e.g. means) or other basic estimates (e.g. regression coefficient) AND variation (e.g. standard deviation) or associated estimates of uncertainty (e.g. confidence intervals)
☐	☒	For null hypothesis testing, the test statistic (e.g. *F*, *t*, *r*) with confidence intervals, effect sizes, degrees of freedom and *P* value noted<br>*Give P values as exact values whenever suitable.*
☒	☐	For Bayesian analysis, information on the choice of priors and Markov chain Monte Carlo settings
☒	☐	For hierarchical and complex designs, identification of the appropriate level for tests and full reporting of outcomes
☒	☐	Estimates of effect sizes (e.g. Cohen's *d*, Pearson's *r*), indicating how they were calculated

Our web collection on statistics for biologists contains articles on many of the points above.

Software and code

Policy information about availability of computer code

Data collection	EPU 2.13, ClampFit 11.3
Data analysis	Ctffind-4.1.10, Relion-5.0, CryoSparc-v4..0, MotionCorr2-1.4.0, Topaz v0.2.4, Phenix v1.21.2-5419, Coot-0.9.8.95, UCSF chimeraX-1.8, PyMol-3.0.4, OriginPro 2024, GraphPad Prism 10

For manuscripts utilizing custom algorithms or software that are central to the research but not yet described in published literature, software must be made available to editors and reviewers. We strongly encourage code deposition in a community repository (e.g. GitHub). See the Nature Portfolio guidelines for submitting code & software for further information.

Data

Policy information about availability of data

All manuscripts must include a data availability statement. This statement should provide the following information, where applicable:

- Accession codes, unique identifiers, or web links for publicly available datasets
- A description of any restrictions on data availability
- For clinical datasets or third party data, please ensure that the statement adheres to our policy

Cryo-EM density maps have been deposited at the EMDB (Electron Microscopy Data Bank) and the Research Collaboratory for Structural Bioinformatics Protein Data Bank (RCS-PDB), respectively. The EMDB accession code and the PDB accession code for Ca2+/TPPO–TRPM4–37°C is EMDB-73754 and PDB 9Z1W; Ca2+/TPPO–TRPM4–37°C warm TMD is EMDB- 73755, PDB 9Z1X; Ca2+/TPPO–TRPM4–37°C cold TMD is EMDB-73756, PDB 9Z1Y; Ca2+/TPPO–TRPM4–18°C is

EMDB-73757, PDB 9Z1Z; EGTA/TPPO–TRPM4– 37°C is EMDB-73758, PDB 9Z20; Ca2+/NC1–TRPM4–37°C is EMDB-73759, PDB 9Z21; EGTA/NC1– TRPM4–37°C is EMDB-73760, PDB 9Z22; Ca2+/CBA–TRPM4–37°C is EMDB-73761, PDB 9Z23; Ca2+/CBA–TRPM4–37°C TMD is EMDB-73762, PDB 9Z24; Ca2+/NBA–TRPM4–37°C is EMDB-73763, PDB 9Z25; Ca2+/NBA–TRPM4–37°C TMD is EMDB-73764, PDB 9Z26; Ca2+/CBA/DVT–TRPM4–37°C is EMDB-73765, PDB 9Z27; respectively.

Research involving human participants, their data, or biological material

Policy information about studies with human participants or human data. See also policy information about sex, gender (identity/presentation), and sexual orientation and race, ethnicity and racism.

Reporting on sex and gender	Not applicable
Reporting on race, ethnicity, or other socially relevant groupings	Not applicable
Population characteristics	Not applicable
Recruitment	Not applicable
Ethics oversight	Not applicable

Note that full information on the approval of the study protocol must also be provided in the manuscript.

Field-specific reporting

Please select the one below that is the best fit for your research. If you are not sure, read the appropriate sections before making your selection.

☒ Life sciences ☐ Behavioural & social sciences ☐ Ecological, evolutionary & environmental sciences

For a reference copy of the document with all sections, see nature.com/documents/nr-reporting-summary-flat.pdf

Life sciences study design

All studies must disclose on these points even when the disclosure is negative.

Sample size	The sample sizes of the cryo-EM data were not predetermined, but were determined/limited by the available time of the microscope. These sample sizes were large enough to allow for the reconstruction of cryo-EM maps of sufficiently high quality to draw the conclusions of this study.
Data exclusions	During cryo-EM data processing, particles that clearly did not show target protein-like feature or showed broken/disordered protein domain(s) were excluded, consistent with common practice in the field.
Replication	For electrophysiology experiments, the number of biologically independent experimental replicates/measurements are indicated in the figure legend. For cryo-EM, all attempts to replicate the structural findings were successful.
Randomization	This study did not allocate experimental groups. Therefore, no randomization was necessary.
Blinding	Blinding was not applicable to electrophysiology or cryo-EM studies.

Reporting for specific materials, systems and methods

We require information from authors about some types of materials, experimental systems and methods used in many studies. Here, indicate whether each material, system or method listed is relevant to your study. If you are not sure if a list item applies to your research, read the appropriate section before selecting a response.

Materials & experimental systems

n/a	Involved in the study	
☒	☐	Antibodies
☐	☒	Eukaryotic cell lines
☒	☐	Palaeontology and archaeology
☒	☐	Animals and other organisms
☒	☐	Clinical data
☒	☐	Dual use research of concern
☒	☐	Plants

Methods

n/a	Involved in the study	
☒	☐	ChIP-seq
☒	☐	Flow cytometry
☒	☐	MRI-based neuroimaging

Eukaryotic cell lines

Policy information about cell lines and Sex and Gender in Research

Cell line source(s)	The Gibco Sf9 cells (Catalog 12-659-017) are obtained from Fisher Scientific. The tsA201 cells (Catalog CRL-3216) are obtained from ATCC.
Authentication	The cells were purchased from commercial sources and routinely maintained in our lab. They were not independently authenticated for these studies.
Mycoplasma contamination	sf9 cells and tsA201 cells were tested negative for Mycoplasma contamination.
Commonly misidentified lines (See ICLAC register)	No commonly misidentified lines were used.

Plants

Seed stocks	Not applicable
Novel plant genotypes	Not applicable
Authentication	Not applicable
