# Supplementary material for: Temperature and intrinsic Ca2+ reshape TRPM4 pharmacology
Source: Nat Struct Mol Biol. 2026 Jun 9;33(6):973–84. doi: 10.1038/s41594-026-01818-3 (PMC13275316; doi:10.1038/s41594-026-01818-3)
Supplement: Supplementary file 7 — Uncropped gel in Fig. 4c. [file 41594_2026_1818_MOESM7_ESM.pdf]

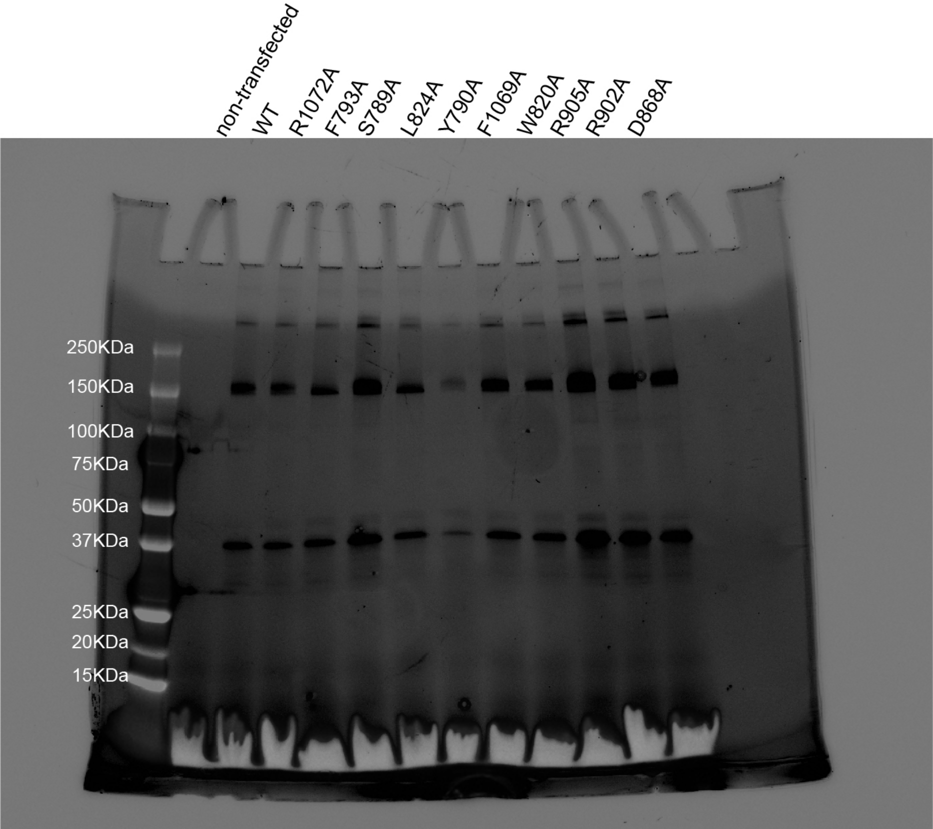
non-transfected
WT
R1072A
F793A
S789A
L824A
Y790A
F1069A
W820A
R905A
R902A
D868A
250KDa
150KDa
100KDa
75KDa
50KDa
37KDa
25KDa
20KDa
15KDa

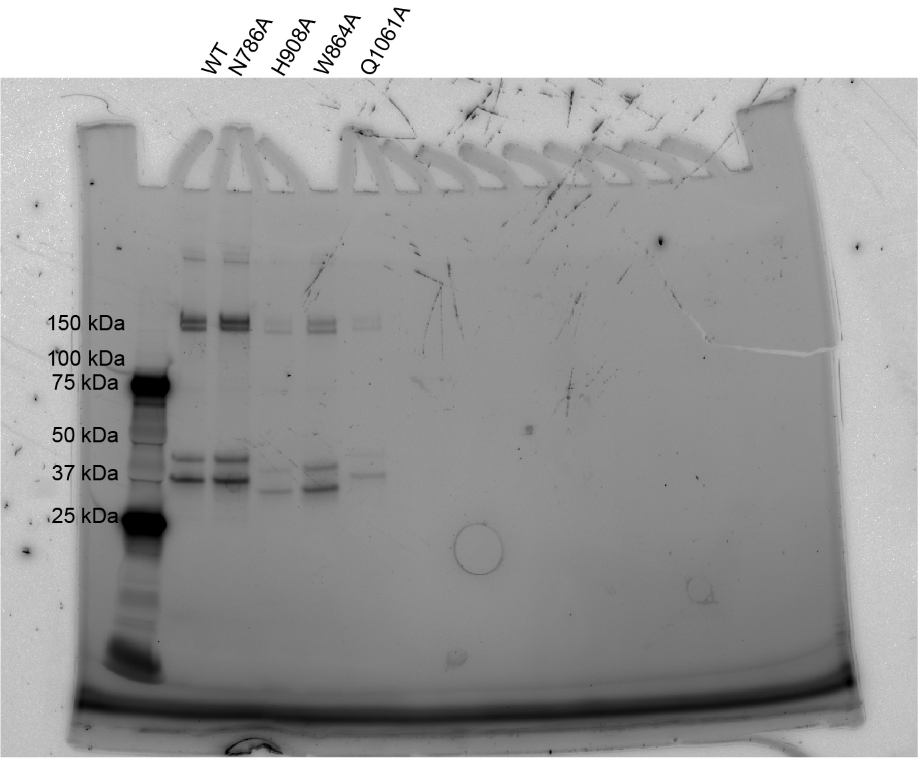
WT
N786A
H908A
W864A
Q1061A
150 kDa
100 kDa
75 kDa
50 kDa
37 kDa
25 kDa
