# Supplementary figures and images for: Temperature and intrinsic Ca2+ reshape TRPM4 pharmacology
Source: Nat Struct Mol Biol. 2026 Jun 9;33(6):973–84. doi: 10.1038/s41594-026-01818-3 (PMC13275316; doi:10.1038/s41594-026-01818-3)

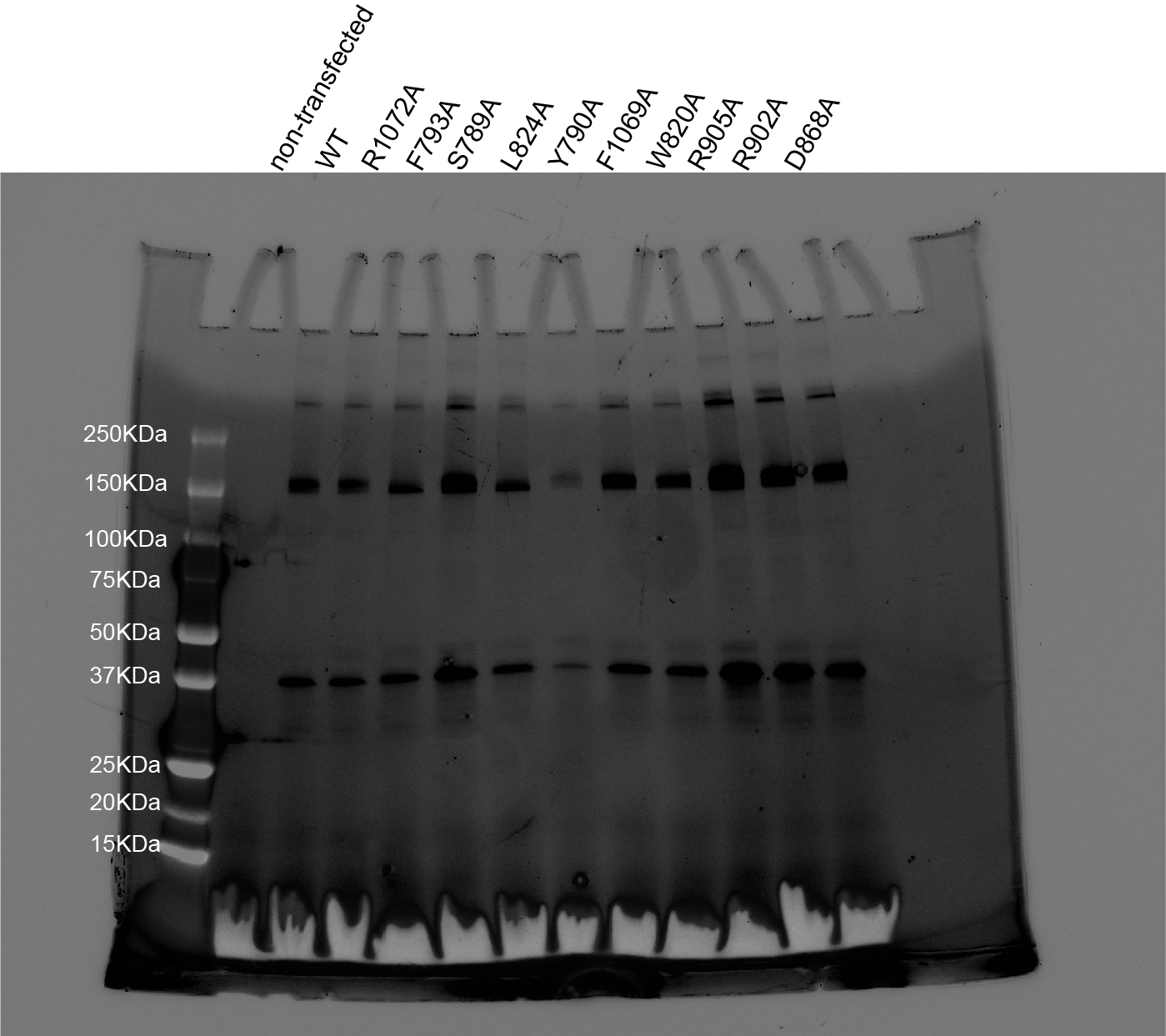

Supplement: Supplementary file 10 — Uncropped gel in Extended Data Fig. 1j. [file 41594_2026_1818_MOESM10_ESM.jpg]
