# Supplementary material for: Temperature and intrinsic Ca2+ reshape TRPM4 pharmacology
Source: Nat Struct Mol Biol. 2026 Jun 9;33(6):973–84. doi: 10.1038/s41594-026-01818-3 (PMC13275316; doi:10.1038/s41594-026-01818-3)
Supplement: Supplementary file 3 — Peer Review File [file 41594_2026_1818_MOESM3_ESM.pdf]

nature portfolio

# Peer Review File

# Temperature and intrinsic Ca2+ reshape TRPM4 pharmacology

Corresponding Author: Dr Wei Lü

Version 0:

Decision Letter:

13th Jan 2026

Dear Dr. Lü,

Thank you again for submitting your manuscript "Temperature and intrinsic Ca2+ reshape TRPM4 pharmacology". I apologize for the delay in sharing our decision. We have decided to send your manuscript for peer review, but several points require your attention before we can proceed with peer review.

I am re-opening the manuscript submission system for you to resubmit your manuscript with all associated files needed for peer review directly, within 2-3 business days if possible. Please follow the link at the bottom of this email to upload the documents listed below. If you have any issues, please reach out to us before completing the submission.

1. STRUCTURAL DATA:
We require official wwPDB validation reports for newly described atomic structures per journal policy. We also request that authors provide cryo-EM maps, half-maps and models, as well as maps and models obtained from subtomogram averaging if it applies to the work, to help the reviewers in assessing the work. We recommend the use of figshare in our system, which allows for provision of anonymous access links for the referees (https://www.springernature.com/gp/authors/research-data/figshare-integration). Alternatively, please upload .zip folders directly with the submission.
Manuscripts reporting new structures should contain a table summarizing structural and refinement statistics. Please include these tables for new cryo-EM or -ET (https://www.nature.com/documents/nr-tables-cryo-em.doc and modifying this file for ET), NMR (https://www.nature.com/documents/nr-tables-nmr.doc) and X-ray crystallography (https://www.nature.com/documents/nr-tables-xray.doc). To facilitate assessment of the quality of the structural data, a stereo image of a portion of the electron density map (for crystallography papers) or of the superimposed lowest energy structures (>10; for NMR papers) should be provided with the submitted manuscript. If the reported structure represents a novel overall fold, a stereo image of the entire structure (as a backbone trace) should be provided. For cryo-EM structures, a representative micrograph showing individual particles should be provided in the figures alongside a processing workflow (please see our Editorial for more information: https://www.nature.com/articles/s41594-025-01567-9 and here for an example: https://www.nature.com/articles/s41594-025-01586-6/figures/7).

2. REPORTING SUMMARY:
We want to ensure that the methods and statistics reporting in our papers are of the highest quality. To that end, we ask authors to fill out a Reporting Summary that collects information on experimental design and reagents. If your paper includes ChIP-seq or flow cytometry data, we ask that you take special care to complete those sections of the Reporting Summary as this data will aid greatly in the review of your manuscript.

Reporting Summary:
https://www.nature.com/documents/nr-reporting-summary.pdf

3- PROTEOMICS AND SEQUENCING:
Please provide accession numbers and reviewer tokens to access sequencing or proteomics datasets if any unpublished datasets are part of your study. Please add this information to your manuscript file. Proteomics data should be deposited to any ProteomeXchange member repository (http://www.proteomexchange.org/) and DNA sequencing, RNA sequencing, genome assembly data should be deposited to any INSDC member repository (https://www.insdc.org/) or Genome Sequence Archive.

4- CODE, MD SIMULATIONS, AND MACHINE LEARNING REPORTING:

Please provide the code used to analyse the data if newly developed (unpublished) code was used in the work. For the reviewers to evaluate the work adequately, they must be able to test the software/review the code themselves. If you have not yet provided the software, we therefore request that you provide a single compressed zip file containing the software with a readme.txt file or other user manual containing complete instructions for installing and running the software. If appropriate, please provide example data and expected output. Sufficient material should be provided for referees to directly test the performance of the software/algorithm. Alternatively, you can upload the code to GitHub and provide the link directly in the manuscript.

Please fill out and upload the code and software submission checklist that will be made available to editors and reviewers. This form is a dynamic 'smart pdf' and must therefore be downloaded and completed in Adobe Reader, instead of opening it in a web browser.
https://www.nature.com/documents/nr-software-policy.pdf

If molecular dynamics (MD) simulations were performed, please refer to this Editorial and provide a completed MD simulations checklist with your manuscript: https://www.nature.com/articles/s42003-023-04653-0

Lastly, if it applies, please complete the completed machine learning checklist with your submission and upload it for review: https://www.nature.com/documents/machine-learning-checklist.pdf

Please use the link below to submit the files. **Please also remember to move forward all other files associated with this version of the paper.**

Link Redacted

**Note:** This URL links to your confidential home page and associated information about manuscripts you may have submitted, or that you are reviewing for us. If you wish to forward this email to co-authors, please delete the link to your homepage.

We look forward to seeing the revised manuscript and thank you for the opportunity to review your work.

Sincerely,
Kat

Katarzyna Ciazynska, PhD
(she/her)
Senior Editor
Nature Structural & Molecular Biology
https://orcid.org/0000-0002-9899-2428

Version 1:

Decision Letter:

13th Feb 2026

Dear Dr. Lü,

Thank you again for submitting your manuscript "Temperature and intrinsic Ca2+ reshape TRPM4 pharmacology" to the journal. I apologize for the delay in responding, which resulted from the difficulty in obtaining referee reports. We now have comments (below) from the 3 reviewers who evaluated your paper. In light of those reports, we remain interested in your study and would like to see your response to the comments of the referees in the form of a revised manuscript.

You will see that while reviewers appreciate the results, they raise several concerns which will need to be addressed in a revision. Specifically, we ask that you provide all clarifications and discussion points requested by the reviewers, especially those regarding NC1 and Ca2+ binding. We agree that in absence of data to support the generalisability of the model, those claims should be toned down. Please provide all the controls requested by reviewer the reviewrs. Reviewer's #3 requests for additional mutagenesis experiments would also be of interest, if feasible to perform within the scope of revision.

Please be sure to address/respond to all concerns of the referees in full in a point-by-point response and highlight all changes in the revised manuscript text file. We expect to see your revised manuscript within 6-8 weeks. We are committed to providing a fair and constructive peer-review process; please do not hesitate to contact us if you would like to discuss the reviews or anticipate any delay with the resubmission. If you cannot send it within this time, please contact us to discuss an extension; we will still consider your revision provided that no similar work has been accepted for publication at NSMB or published elsewhere.

REPORTING AND DATA AVAILABILITY: Please provide updated files as follows with your revision. As you already know, we put great emphasis on ensuring that the methods and statistics reported in our papers are correct and accurate.

1. REPORTING SUMMARY: Please provide an updated file:

https://www.nature.com/documents/nr-reporting-summary.pdf

This form is a dynamic ‘smart pdf’ and must be downloaded and completed in Adobe Reader.

2. REPORTING OF STRUCTURAL DATA: If there are additional or modified structures in the revision, please submit the corresponding PDB validation reports with your other files, and use the figshare integration system to provide access to maps, half-maps, and models.

Manuscripts reporting new structures should contain a table summarizing structural and refinement statistics. Please include these tables for new cryo-EM or -ET (https://www.nature.com/documents/nr-tables-cryo-em.doc and modifying this file for ET), NMR (https://www.nature.com/documents/nr-tables-nmr.doc) and X-ray crystallography (https://www.nature.com/documents/nr-tables-xray.doc). To facilitate assessment of the quality of the structural data, a stereo image of a portion of the electron density map (for crystallography papers) or of the superimposed lowest energy structures (>10; for NMR papers) should be provided with the submitted manuscript. If the reported structure represents a novel overall fold, a stereo image of the entire structure (as a backbone trace) should also be provided. For cryo-EM structures, a representative micrograph showing individual particles should be provided in the figures alongside a processing workflow (please see our Editorial for more information: https://www.nature.com/articles/s41594-025-01567-9).

DATA DEPOSITION: We require deposition of coordinates (and, in the case of crystal structures, structure factors) into the Protein Data Bank with the designation of immediate release upon publication (HPUB). Electron microscopy-derived density maps and coordinate data must be deposited in EMDB and released upon publication. Deposition and immediate release of NMR chemical shift assignments are requested. To avoid delays in publication, dataset accession numbers must be supplied with the revised manuscript and appropriate release dates must be indicated at the galley proof stage.

3. REPORTING OF LIGHT MICROSCOPY DATA: For any revision that includes light microscopy data, we ask our authors to please include a completed light microscopy reporting table [https://www.nature.com/documents/Light_microscopy_reporting_table.xlsx] to ensure the methods are described thoroughly. The table will be available to reviewers and

ultimately published, should the manuscript be accepted at the journal. When submitting the revised version of your manuscript, please pay close attention to our href="https://www.nature.com/nature-portfolio/editorial-policies/image-integrity">Digital Image Integrity Guidelines.</a>

4. CODE AND COMPUTATIONAL WORK: If newly developed, unpublished code was used in this work, it must be provided with the revision and access to it must be disclosed in the Code Availability Statement in the manuscript. Please also provide the completed software submission checklist. This form is a dynamic ‘smart pdf’ and must be downloaded and completed in Adobe Reader, instead of opening it in a web browser: https://www.nature.com/documents/nr-software-policy.pdf

If molecular dynamics (MD) simulations were performed, please refer to this Editorial and provide a completed MD simulations checklist with your revision: https://www.nature.com/articles/s42003-023-04653-0

Lastly, if it applies, please complete and upload the completed machine learning checklist with your submission for re-review: https://www.nature.com/documents/machine-learning-checklist.pdf

5. SOURCE GEL IMAGES: Unprocessed scans should be provided for all gels and western blots presented in figures and should be clearly labelled with the figure number in the file title. The source gel images should be uncropped, unmodified images, with molecular weight markers. Uncropped, unmodified gel images from existing experimental repeats, in addition to the data shown in the figures, can also be presented in source data. Please provide these files as PDF with your revision.

Please ensure that all control panels for gels and western blots are appropriately described as loading or IP controls. Lastly, please ensure that all images in the paper are checked for duplication of panels and for splicing of gel lanes.

6. SOURCE NUMERICAL DATA: we urge authors to provide, in tabular form in excel files, the data underlying the graphical representations used in figures. This is to further increase transparency in data reporting, as detailed in this editorial (http://www.nature.com/nsmb/journal/v22/n10/full/nsmb.3110.html). Please provide one excel file per figure max, with one panel per tab. When submitting files, the title field should indicate which figure the source data pertains to.

7. DATA AVAILABILITY: this journal strongly supports public availability of data. All data used in accepted papers should be available via a public data repository, or alternatively, as Supplementary Information. If data can only be shared on request, please explain why in your Data Availability Statement and in the correspondence with your editor.

For some data types, deposition in a public repository is mandatory as detailed below:

https://www.nature.com/nature-research/editorial-policies/reporting-standards#availability-of-data

Finally, please ensure that you retain unprocessed data and metadata files after publication, ideally archiving data in perpetuity, as these may be requested during the peer review and production process or after publication if any issues arise.

EXTENDED DATA FIGURES

When re-submitting your manuscript, please ensure that any supplementary figures and tables that are crucial to the manuscript's conclusions are converted into Extended Data figures and tables to increase visibility of these data. Extended Data figures and tables are online-only (present in the online PDF and full-text HTML versions of the paper), peer-reviewed display items that provide essential background to the article but are not included in the main article due to space constraints. A maximum of ten Extended Data display items (figures and tables) is permitted.

While we encourage the use of color in preparing figures, please note that this will incur a charge to partially defray the cost of printing. Information about color charges can be found at http://www.nature.com/nsmb/authors/submit/index.html#costs

Nature Structural & Molecular Biology is committed to improving transparency in authorship. As part of our efforts in this direction, we are now requesting that all authors identified as 'corresponding author' on published papers create and link their Open Researcher and Contributor Identifier (ORCID) with their account on the Manuscript Tracking System (MTS), prior to acceptance. This applies to primary research papers only. ORCID helps the scientific community achieve unambiguous attribution of all scholarly contributions. You can create and link your ORCID from the home page of the MTS by clicking on 'Modify my Springer Nature account'. For more information please visit please visit <a href="http://www.springernature.com/orcid">www.springernature.com/orcid</a>.

Please use the link below to submit your revised manuscript and related files:

Link Redacted

**Note:** This URL links to your confidential home page and associated information about manuscripts you may have submitted, or that you are reviewing for us. If you wish to forward this email to co-authors, please delete the link to your homepage.

We look forward to seeing the revised manuscript and thank you for the opportunity to review your work.

Sincerely,

Katarzyna Ciazynska, PhD
(she/her)
Senior Editor
Nature Structural & Molecular Biology
https://orcid.org/0000-0002-9899-2428

Referee expertise:

Referee #1: cryo-EM, membrane proteins

Referee #2: TRP channels, structural biology

Referee #3: TRP channels

Reviewers' Comments:

Reviewer #1 (Remarks to the Author):

This manuscript by Hu et al. reports structures of TRPM4 in complex with various small molecules under different $Ca^{2+}$ concentrations and temperatures. Together with robust electrophysiological recordings, the authors reveal a complex

interplay among small-molecule modulators, $Ca^{2+}$, and temperature in controlling TRPM4 gating. The work substantially advances our understanding of TRPM4 regulation and pharmacology and, more broadly, underscores the importance of evaluating channel structure, function, and drug responses under physiologically relevant conditions. I have the following minor questions and suggestions that may help further clarify and strengthen the manuscript:
1. Line 110. In reference 34, while the electrophysiology experiments were conducted at room temperature, I could not find any indication that the fluorescence-based membrane potential assays in that study were performed at room temperature. Could the authors clarify this? To my knowledge, FLIPR or other fluorescence-based membrane potential assays are often conducted at 37°C, as plate readers from Molecular Devices can maintain temperature 37°C.
2. Line 142. The authors state "as both produce the hallmark outwardly rectified currents." Could the authors clarify what "both" refers to here? In Fig. 1f, the +TPPO current appears relatively linear rather than strongly outwardly rectified.
3. Line 191. In Extended Data Fig. 1k, the currents of R1072A with and without TPPO should be shown and compared, rather than comparing WT versus R1072A.
4. Line 195. Could the authors comment on what happens to the $Ca^{2+}$-free warm conformation in this dataset? Does this conformation not exist under this particular condition?
5. If both warm and cold conformations are present within a given dataset, I suggest that the authors clearly label whether each figure represents the warm or cold conformation. This could be indicated directly on the figures (e.g., Fig. 3c, 4a, 4b, 5a, and 5b) to improve the clarity.
6. Lines 247–249. The authors provide one possible explanation, but this does not rule out the possibility that NC1 and $Ca^{2+}$ can bind simultaneously. NC1 affinity may decrease in the presence of $Ca^{2+}$, but this does not necessarily mean that their binding is mutually incompatible. The electrophysiological phenotype could be explained by $Ca^{2+}$ displacing NC1, but it could also reflect co-binding of $Ca^{2+}$ and NC1 producing a distinct electrophysiological profile.
7. Line 376. At elevated $Ca^{2+}$, does NC1 truly become antagonized, or does it simply have minimal effect (become neutral)? In Fig. 2c and 2f, it is difficult to determine whether NC1 has any positive or negative effect under these conditions.
8. Could the authors briefly discuss the recent finding that NC1 activation may depend on $PIP_2$ (https://doi.org/10.64898/2026.01.28.702369)? How does this integrate into the model presented in Fig. 6?

Reviewer #2 (Remarks to the Author):

In this elegant manuscript by Hu et al., the authors evaluated the influence of two endogenous factors, temperature and intracellular Ca2+, on the pharmacological regulation of ion channel TRPM4. By combining electrophysiological recordings with structural analyses by cryo-EM, the authors made several novel findings: 1) TPPO, a ligand that is considered to be inactive with TRPM4, demonstrated a robust activation effect under physiological temperature and intracellular Ca2+; 2) NC1 functions as an activator only when the intracellular Ca2+ is low; and 3) NBA and CBA inhibit TRPM4 clearly via a site that is different from the one previously reported. This manuscript presents strong cases demonstrating the interplay between commonly overlooked endogenous factors, such as temperature, and the pharmacological profiles of ligands, with profound implications for a broad range of scientific fields studying ligand-receptor interactions and their translational applications. This manuscript also revealed the role of the conformational equilibrium of the "cold" and "warm" states, as well as the Ca2+ binding states in the TMD site, in defining the pharmacological outcomes of ligands, which have a strong impact on our structural understanding of TRPM ion channel gating. I have a few comments that could potentially improve the manuscript.
1. In Lines 135-139, the authors discuss the change in potency of TPPO by altering the temperature and intracellular Ca2+. Based on Figure 1, under the assumption that the expression levels are comparable in different conditions, there is also a large change in efficacy. Is the assumption about the expression true? If so, it would be interesting to discuss the potential mechanisms underlying the change in efficacy.
2. In Fig. 1f, there is a clear partial loss of the outward rectification that is characteristic at room temperature or basal Ca2+ under 37 degree. This is similar to the current elicited by NC1. In the previous work, it appears that the strongly rectifying current is associated with Ca2+-mediated activation, and the weakly rectifying current is Ca2+-independent. I think this is an interesting observation that the authors could point out and potentially discuss if there is any hypothesis regarding the mechanism.
3. In Fig 3d, it may be better to have the coordinates of the ligand with the map in the cold state as well to illustrate the difference in how strong the map intensity is with respect to the ligand.
4. In Extended Data Fig. 1 j and k, are the current levels normalized to the expression levels of the mutants? In addition, I may have missed it, but where is the data on the TPPO activation of the mutants other than R1072A?
5. In Line 200, "ref" should be deleted.
6. Similar to point 4, for the mutants in Fig. 4C, are their currents normalized by their expression level?
7. In Lines 247-250, that authors stated that "The weak residual density, likely arising from the oversaturating NC1 concentration used for cryo-EM rather than genuine co-binding, supports that NC1 and Ca2+ binding are mutually incompatible." This statement appears to be overly strong to me. I agree with the authors that this is a very likely scenario and that cryo-EM data processing may not be able to perfectly separate molecules into classes with different occupancies. However, there is no direct evidence supporting this claim. It is probably better to leave other possibilities open here.

Reviewer #3 (Remarks to the Author):

Revisiting the minimal established knowledge of TRPM4 pharmacology through the lens of physiological temperature has clearly been a thoughtful and enlightening endeavor. The authors have identified TPPO as a TRPM4 activator whose activity is restricted to physiologically relevant temperatures and calcium concentrations. Whereas the agonist NC1 loses its

potency at high calcium. They also observe that, although both agonists and antagonists bind within the S1–S4 bundle, they occupy distinct sites (ie. S1-S4high for agonists and S1-S4low for antagonists). The authors use patch-clamp electrophysiology, single particle cryo-EM, and structure-function analysis for this study. The findings are original, rigorous, and well presented. Overall, the data quality is good, and the conclusions are consistent with the data. Furthermore, the results presented are of interest to those in the TRP channel, broader ion channel, and potential protein biochemistry fields.

Overall, I question the strong emphasis placed on the broad applicability of these findings (i.e., that temperature and calcium influence TRPM4 pharmacology) to proteins in general. As written, the manuscript does not present evidence supporting this generalization. For example, were similar approaches applied to other ion channels, and if so, did they yield comparable insights? If additional examples exist, incorporating them into the introduction or discussion would strengthen this claim. Alternatively, if such data are not available, I suggest tempering the claim accordingly and adding a statement in the discussion noting that testing the broader applicability of these observations would be an important avenue for future work.

I leave the following suggestions and comments for the authors consideration.

The introduction and discussion emphasize the importance of physiological temperature and calcium concentrations for mechanistic protein studies but gives comparatively little attention to the biological role of TRPM4. This emphasis feels somewhat unbalanced, and I suggest reorganizing these paragraphs to more clearly highlight TRPM4’s physiological relevance.

The authors should better explain what is meant by Brugada syndrome and inherited conduction disorders, particularly for readers who may not be familiar with these conditions. The authors should also clarify whether TRPM4 agonists or antagonists are likely to be relevant for treating these diseases.

Neither the text nor the figures link ligand binding, whether by activators or inhibitors, to structural changes in the pore. In the absence of the underlying maps and models, this aspect of the story remains missing. I suggest that this be remedied by explicitly addressing pore conformational changes. This will strengthen the completeness of the mechanistic interpretation.

Using your mutants, is it possible to generate channels that respond to NBA/CBA but not TPPO or NC1, and vice versa? This would help address how linked the pockets are.

The discussion of DVT position modulation lacks sufficient introduction to explain what it is or why this experiment is informative.

Line 365: Change the work protein to TRPM4. Is there another example of a protein whose ligand binding is modulated by temperature and/or calcium?

Minor Comment:

Lines 180-181: Does this sentence have the intended meaning if you remove the word ‘where’?

Version 2:

Decision Letter:

Our ref: NSMB-A52236B

1st Apr 2026

Dear Dr. Lü,

Thank you for submitting your revised manuscript "Temperature and intrinsic Ca2+ reshape TRPM4 pharmacology" (NSMB-A52236B). It has now been seen by the original referees and their comments are below. The reviewers find that the paper has improved in revision, and therefore we'll be happy in principle to publish it in Nature Structural & Molecular Biology, pending minor revisions to satisfy the referees' final requests and to comply with our editorial and formatting guidelines.

You will see that while reviewer #1 did not include any comments, we can confirm they have not raised any further point and are satisfied with the extent of revisions.

We are now performing detailed checks on your paper and will send you a checklist detailing our editorial and formatting requirements in about a week. Please do not upload the final materials and make any revisions until you receive this additional information from us.

To facilitate our work at this stage, it is important that we have a copy of the main text as a word file. If you could please send along a word version of this file as soon as possible, we would greatly appreciate it; please make sure to copy the NSMB account (cc'ed above).

Thank you again for your interest in Nature Structural & Molecular Biology Please do not hesitate to contact me if you have any questions.

Sincerely,
Kat

Katarzyna Ciazynska, PhD
(she/her)
Senior Editor
Nature Structural & Molecular Biology
https://orcid.org/0000-0002-9899-2428

Reviewer #2 (Remarks to the Author):

The authors addressed all my concerns and this manuscript is now suitable for publication.

Reviewer #3 (Remarks to the Author):

The authors have sufficiently addressed this reviewer's comments.

Version 3:

Decision Letter:

30th Apr 2026

Dear Dr. Lü,

We are now happy to accept your revised paper "Temperature and intrinsic Ca2+ reshape TRPM4 pharmacology" for publication as an Article in Nature Structural & Molecular Biology.

Acceptance is conditional on the manuscript's not being published elsewhere and on there being no announcement of this work to the newspapers, magazines, radio or television until the publication date in Nature Structural & Molecular Biology.

Over the next few weeks, your paper will be copyedited to ensure that it conforms to Nature Structural & Molecular Biology style. Once your paper is typeset, you will receive an email with a link to choose the appropriate publishing options for your paper and our Author Services team will be in touch regarding any additional information that may be required.

After the grant of rights is completed, you will receive a link to your electronic proof via email with a request to make any corrections within 48 hours. If, when you receive your proof, you cannot meet this deadline, please inform us at rjsproduction@springernature.com immediately.

You will not receive your proofs until the publishing agreement has been received through our system.

Due to the importance of these deadlines, we ask that you please let us know now whether you will be difficult to contact over the next month. If this is the case, we ask you provide us with the contact information (email, phone and fax) of someone who will be able to check the proofs on your behalf, and who will be available to address any last-minute problems.

To assist our authors in disseminating their research to the broader community, our SharedIt initiative provides all co-authors with the ability to generate a unique shareable link that will allow anyone (with or without a subscription) to read the published article. Recipients of the link with a subscription will also be able to download and print the PDF.

As soon as your article is published, you can generate your shareable link by entering the DOI of your article here: <a href="http://authors.springernature.com/share">http://authors.springernature.com/share</a>. Corresponding authors will also receive an automated email with the shareable link

Note the policy of the journal on data deposition: http://www.nature.com/authors/policies/availability.html.

Your paper will be published online soon after we receive proof corrections and will appear in print in the next available issue. You can find out your date of online publication by contacting the production team shortly after sending your proof corrections.

You may wish to make your media relations office aware of your accepted publication, in case they consider it appropriate to organize some internal or external publicity. Once your paper has been scheduled you will receive an email confirming the

publication details. This is normally 3-4 working days in advance of publication. If you need additional notice of the date and time of publication, please let the production team know when you receive the proof of your article to ensure there is sufficient time to coordinate. Further information on our embargo policies can be found here: https://www.nature.com/authors/policies/embargo.html

You can now use a single sign-on for all your accounts, view the status of all your manuscript submissions and reviews, access usage statistics for your published articles and download a record of your refereeing activity for the Nature journals.

If you have not already done so, we strongly recommend that you upload the step-by-step protocols used in this manuscript to the Protocol Exchange. Protocol Exchange is an open online resource that allows researchers to share their detailed experimental know-how. All uploaded protocols are made freely available, assigned DOIs for ease of citation and fully searchable through nature.com. Protocols can be linked to any publications in which they are used and will be linked to from your article. You can also establish a dedicated page to collect all your lab Protocols. By uploading your Protocols to Protocol Exchange, you are enabling researchers to more readily reproduce or adapt the methodology you use, as well as increasing the visibility of your protocols and papers. Upload your Protocols at www.nature.com/protocolexchange/. Further information can be found at www.nature.com/protocolexchange/about.

An online order form for reprints of your paper is available at <a href="https://www.nature.com/reprints/author-reprints.html">https://www.nature.com/reprints/author-reprints.html</a>. Please let your coauthors and your institutions' public affairs office know that they are also welcome to order reprints by this method.

**Authors may need to take specific actions to achieve compliance with funder and institutional open access mandates.** If your research is supported by a funder that requires immediate open access (e.g. according to <a href="https://www.springernature.com/gp/open-science/plan-s-compliance"> Plan S principles</a> or the <a href="https://www.springernature.com/gp/open-science/us-federal-agency-compliance"> NIH public access policy</a>) then you should select the gold OA route, and we will direct you to the compliant route where possible. Because authors warrant under our subscription licensing terms that they haven't committed to licensing any version of their article under a licence inconsistent with the terms of our agreement – including the applicable embargo period – publication under the subscription model isn't suitable for authors whose funders require no embargo.

In approximately 10 business days you will receive an email with a link to choose the appropriate publishing options for your paper and our Author Services team will be in touch regarding any additional information that may be required.

You will not receive your proofs until the publishing agreement has been received through our system.

If you have any questions about our publishing options, costs, Open Access requirements, or our legal forms, please contact ASJournals@springernature.com

Sincerely,

Katarzyna Ciazynska, PhD
(she/her)
Senior Editor
Nature Structural & Molecular Biology
https://orcid.org/0000-0002-9899-2428

Click here if you would like to recommend Nature Structural & Molecular Biology to your librarian: http://www.nature.com/subscriptions/recommend.html#forms

**Open Access** This Peer Review File is licensed under a Creative Commons Attribution 4.0 International License, which permits use, sharing, adaptation, distribution and reproduction in any medium or format, as long as you give appropriate credit to the original author(s) and the source, provide a link to the Creative Commons license, and indicate if changes were made.
In cases where reviewers are anonymous, credit should be given to 'Anonymous Referee' and the source.
The images or other third party material in this Peer Review File are included in the article's Creative Commons license, unless indicated otherwise in a credit line to the material. If material is not included in the article's Creative Commons license and your intended use is not permitted by statutory regulation or exceeds the permitted use, you will need to obtain permission directly from the copyright holder.
To view a copy of this license, visit https://creativecommons.org/licenses/by/4.0/

*Reviewers' Comments:*

*Reviewer #1 (Remarks to the Author):*

*This manuscript by Hu et al. reports structures of TRPM4 in complex with various small molecules under different $Ca^{2+}$ concentrations and temperatures. Together with robust electrophysiological recordings, the authors reveal a complex interplay among small-molecule modulators, $Ca^{2+}$, and temperature in controlling TRPM4 gating. The work substantially advances our understanding of TRPM4 regulation and pharmacology and, more broadly, underscores the importance of evaluating channel structure, function, and drug responses under physiologically relevant conditions. I have the following minor questions and suggestions that may help further clarify and strengthen the manuscript:*

*1. Line 110. In reference 34, while the electrophysiology experiments were conducted at room temperature, I could not find any indication that the fluorescence-based membrane potential assays in that study were performed at room temperature. Could the authors clarify this? To my knowledge, FLIPR or other fluorescence-based membrane potential assays are often conducted at 37°C, as plate readers from Molecular Devices can maintain temperature 37°C.*

**Response:** We thank the reviewer for pointing this out. We have revised the text to remove "room temperature".

*2. Line 142. The authors state "as both produce the hallmark outwardly rectified currents." Could the authors clarify what "both" refers to here? In Fig. 1f, the +TPPO current appears relatively linear rather than strongly outwardly rectified.*

**Response:** Thanks for this comment. We have previously shown that TRPM4 typically exhibits strong outward rectification at room temperature, regardless of intracellular $Ca^{2+}$ concentration, as well as at physiological temperature under basal intracellular $Ca^{2+}$ conditions. In contrast, at physiological temperature in the presence of high intracellular $Ca^{2+}$, the I–V relationship becomes less outwardly rectifying and can appear more linear (Hu et al., 2024). Although the extent of reduced rectification varies between experimental batches, this trend is consistently observed.

In this context, "both" refers to conditions with and without TPPO in Fig. 1f (37 °C, high $Ca^{2+}$). We note that the current in the presence of TPPO appears relatively linear; however, it is very similar to the current recorded without TPPO, aside from small differences. These minor differences may reflect a combined effect of TPPO and $Ca^{2+}$. Importantly, the key message here is that once intracellular $Ca^{2+}$ reaches activating levels, the effect of TPPO is largely—though not completely—masked. We have revised the text to more accurately describe this phenotype (lines 156–164).

*3. Line 191. In Extended Data Fig. 1k, the currents of R1072A with and without TPPO should be shown and compared, rather than comparing WT versus R1072A.*

**Response:** We thank the reviewer for this suggestion, and agree that comparing R1072A currents in the presence and absence of TPPO provides a more relevant assessment. We have updated Extended Data Fig. 1k to show R1072A currents with and without TPPO, replacing the original WT versus R1072A comparison. The new data unambiguously demonstrate that R1072A is insensitive to TPPO, consistent with our previous conclusion.

*4. Line 195. Could the authors comment on what happens to the $Ca^{2+}$-free warm conformation in this dataset? Does this conformation not exist under this particular condition?*

**Response:** As shown in our previous work (Hu et al., 2024), formation of the warm conformation strictly depends on $Ca^{2+}$ binding at the intracellular site. Consequently, a $Ca^{2+}$-free warm conformation is not observed under this condition.

*5. If both warm and cold conformations are present within a given dataset, I suggest that the authors clearly label whether each figure represents the warm or cold conformation. This could be indicated directly on the figures (e.g., Fig. 3c, 4a, 4b, 5a, and 5b) to improve the clarity.*

**Response:** To improve clarity, we have added explicit labels indicating the "warm" and "cold" conformations directly on the relevant figures, including Figures 3c, 4a, 4b, 5a, and 5b.

*6. Lines 247–249. The authors provide one possible explanation, but this does not rule out the possibility that NC1 and $Ca^{2+}$ can bind simultaneously. NC1 affinity may decrease in the presence of $Ca^{2+}$, but this does not necessarily mean that their binding is mutually incompatible. The electrophysiological phenotype could be explained by $Ca^{2+}$ displacing NC1, but it could also reflect co-binding of $Ca^{2+}$ and NC1 producing a distinct electrophysiological profile.*

**Response:** We agree that our data do not formally rule out the possibility of co-binding of $Ca^{2+}$ and NC1. However, our electrophysiological data show that NC1 has a negligible effect at 1 μM $Ca^{2+}$, and our structural data provide direct evidence that NC1 binding is significantly reduced in the presence of $Ca^{2+}$. We have therefore rephrased this section to acknowledge both possibilities: that the observed electrophysiological phenotype is most consistent with a $Ca^{2+}$-dependent reduction, or near abolition, of NC1 binding, reflecting incompatibility, while also noting that co-binding could, in principle, occur and produce an electrophysiological profile similar to $Ca^{2+}$ alone. We further discuss why the former interpretation is more likely in light of our structural findings (lines 269–280).

*7. Line 376. At elevated $Ca^{2+}$, does NC1 truly become antagonized, or does it simply have minimal effect (become neutral)? In Fig. 2c and 2f, it is difficult to determine whether NC1 has any positive or negative effect under these conditions.*

**Response:** This comment is closely related to comment #6. Based on our structural data, antagonism is indeed the most likely explanation at elevated $Ca^{2+}$ concentrations, in the sense that $Ca^{2+}$ strongly reduces or nearly abolishes NC1 binding. Functionally, this manifests as NC1 having little to no observable effect on the electrophysiological phenotype under these conditions relative to $Ca^{2+}$ alone (Fig. 2c and 2f). We have clarified this distinction in the revised text, emphasizing that the lack of an observable effect of NC1 at high $Ca^{2+}$ is most consistent with $Ca^{2+}$-dependent antagonism at the binding level.

*8. Could the authors briefly discuss the recent finding that NC1 activation may depend on $PIP_2$ (https://doi.org/10.64898/2026.01.28.702369)? How does this integrate into the model presented in Fig. 6?*

**Response:** We appreciate the reviewer for drawing our attention to this recent study reporting PIP2 dependence of NC1 activation. That work was performed using inside-out patch

recordings, a configuration in which PIP2 can be progressively depleted over time. In contrast, our experiments were conducted in the whole-cell configuration, where lipid homeostasis is likely more stably maintained by endogenous cellular processes. This difference in recording configuration could contribute to the preservation of NC1 activity in our experiments, as whole-cell recordings more closely maintain the physiological cellular environment.

At present, our data do not allow us to conclude whether PIP2 binding is strictly required for NC1-mediated activation under physiological conditions. Moreover, this question is not the focus of our study and is not required to explain the $Ca^{2+}$-dependent antagonism of NC1, which is the central focus of our structural and functional analyses. Accordingly, we have not incorporated PIP2 explicitly into the model shown in Fig. 6.

*Reviewer #2 (Remarks to the Author):*

*In this elegant manuscript by Hu et al., the authors evaluated the influence of two endogenous factors, temperature and intracellular Ca2+, on the pharmacological regulation of ion channel TRPM4. By combining electrophysiological recordings with structural analyses by cryo-EM, the authors made several novel findings: 1) TPPO, a ligand that is considered to be inactive with TRPM4, demonstrated a robust activation effect under physiological temperature and intracellular Ca2+; 2) NC1 functions as an activator only when the intracellular Ca2+ is low; and 3) NBA and CBA inhibit TRPM4 clearly via a site that is different from the one previously reported. This manuscript presents strong cases demonstrating the interplay between commonly overlooked endogenous factors, such as temperature, and the pharmacological profiles of ligands, with profound implications for a broad range of scientific fields studying ligand-receptor interactions and their translational applications. This manuscript also revealed the role of the conformational equilibrium of the "cold" and "warm" states, as well as the Ca2+ binding states in the TMD site, in defining the pharmacological outcomes of ligands, which have a strong impact on our structural understanding of TRPM ion channel gating. I have a few comments that could potentially improve the manuscript.*

*1. In Lines 135-139, the authors discuss the change in potency of TPPO by altering the temperature and intracellular Ca2+. Based on Figure 1, under the assumption that the expression levels are comparable in different conditions, there is also a large change in efficacy. Is the assumption about the expression true? If so, it would be interesting to discuss the potential mechanisms underlying the change in efficacy.*

**Response:** We thank the reviewer for this comment. We believe that the apparent "large change in efficacy" reflects a shift in potency rather than a true change in maximal efficacy. Specifically, Fig. 1b, d, and e were all recorded at 50 μM TPPO; however, based on the dose–response curves (Fig. 1g, h and i), this concentration is near-saturating under the condition shown in Fig. 1e but well below the EC50 under the conditions shown in Fig. 1b and d. Consequently, the large current in Fig. 1e and the smaller currents in Fig. 1b and d are expected and do not indicate a change in efficacy.

Regarding expression levels, all recordings were obtained from cells expressing WT TRPM4 under identical experimental conditions. While expression was not quantified at the single-cell level, we would not expect systematic differences in expression between these conditions.

*2. In Fig. 1f, there is a clear partial loss of the outward rectification that is characteristic at room temperature or basal Ca2+ under 37 degree. This is similar to the current elicited by NC1. In the previous work, it appears that the strongly rectifying current is associated with Ca2+-mediated activation, and the weakly rectifying current is Ca2+-independent. I think this is an interesting observation that the authors could point out and potentially discuss if there is any hypothesis regarding the mechanism.*

**Response:** We thank the reviewer for this insightful observation. We have previously reported that TRPM4 typically exhibits strong outward rectification at room temperature, regardless of intracellular $Ca^{2+}$ concentration, as well as at physiological temperature under basal intracellular $Ca^{2+}$ conditions. In contrast, at physiological temperature in the presence of elevated intracellular $Ca^{2+}$, the I–V relationship becomes less outwardly rectifying (Hu et al., 2024). We attribute this change to a temperature- and $Ca^{2+}$-dependent transition of the channel from a "cold" to a "warm" conformation, which requires the combined effects of physiological temperature and $Ca^{2+}$. To make this mechanistic framework clearer to the reader, we have added this explanation to the Introduction (lines 88–93).

*3. In Fig 3d, it may be better to have the coordinates of the ligand with the map in the cold state as well to illustrate the difference in how strong the map intensity is with respect to the ligand.*

**Response:** Thank for the suggestion and we have updated Figure 3d accordingly.

*4. In Extended Data Fig. 1 j and k, are the current levels normalized to the expression levels of the mutants? In addition, I may have missed it, but where is the data on the TPPO activation of the mutants other than R1072A?*

**Response:** We have updated Extended Data Fig. 1j and k to include surface expression data obtained using cell-surface biotinylation followed by in-gel fluorescence imaging (all constructs are GFP-tagged). We intentionally use these expression data qualitatively rather than quantitatively (i.e., we do not normalize currents to expression levels), as normalization could introduce bias given potential uncertainties inherent to this assay.

Even when assessed qualitatively, the expression data support two key conclusions: (1) both WT and all tested mutants traffic to the plasma membrane, and (2) gain-of-function mutants are expressed at levels comparable to or lower than WT, whereas loss-of-function mutants are often expressed at levels comparable to or higher than WT. This qualitative assessment is sufficient to support the functional conclusions presented in the manuscript.

With respect to TPPO sensitivity, we have tested additional mutants beyond R1072A. However, because TPPO is a small ligand and many interacting residues are located near the CaTMD site, mutations at these positions frequently impair $Ca^{2+}$-dependent activation, likely by disrupting the integrity of the adjacent CaTMD site. Since TPPO activity requires an intact CaTMD site, such mutations make it difficult to unambiguously isolate direct effects on TPPO sensitivity (Extended Data Fig. 1j).

For example, the F902A mutant lacks $Ca^{2+}$-dependent activation and is also insensitive to TPPO, making it uninformative for dissecting TPPO-specific effects; accordingly, these data are not included in Extended Data Fig. 1k. As a control mutation that does not affect TPPO sensitivity, we additionally include W820A in Extended Data Fig. 1k. W820A does not impair $Ca^{2+}$ activation and remains responsive to both $Ca^{2+}$ alone and $Ca^{2+}$ plus TPPO. For clarity, we therefore focus on R1072A in the main text, where the effect on TPPO sensitivity can be interpreted unambiguously.

*5. In Line 200, "ref" should be deleted.*

**Response:** We have retained "ref" to prevent potential confusion from placing the citation number directly after "$Ca^{2+}$".

*6. Similar to point 4, for the mutants in Fig. 4C, are their currents normalized by their expression level?*

**Response:** We have updated the figure to include surface expression assay data. Mutants showing reduced or absent NC1-induced currents exhibit surface expression levels comparable to (N786A) or higher than (R905A) WT. The mutant R1072A shows a slightly lower expression level than WT; however, the negligible TPPO effect observed for this mutant (Extended Data Fig. 1k) cannot be explained by this small difference in expression. Together, these data indicate that the loss of NC1-induced current reflects the effects of the mutations rather than impaired trafficking to the cell surface.

*7. In Lines 247-250, that authors stated that "The weak residual density, likely arising from the oversaturating NC1 concentration used for cryo-EM rather than genuine co-binding, supports that NC1 and Ca2+ binding are mutually incompatible." This statement appears to be overly strong to me. I agree with the authors that this is a very likely scenario and that cryo-EM data processing may not be able to perfectly separate molecules into classes with different occupancies. However, there is no direct evidence supporting this claim. It is probably better to leave other possibilities open here.*

**Response:** We appreciate the reviewer's thoughtful feedback. Based on our data, antagonism is the most likely explanation at elevated $Ca^{2+}$ concentrations, in the sense that structurally, $Ca^{2+}$ strongly reduces NC1 binding, and functionally, this manifests as NC1 having little to no observable effect on the electrophysiological phenotype under these conditions relative to $Ca^{2+}$ alone (Fig. 2c and 2f).

Nevertheless, we agree that our original wording was overly definitive. We have therefore rephrased the text to acknowledge alternative possibilities, noting that the observed electrophysiological phenotype and residual density are most consistent with a $Ca^{2+}$-dependent reduction, or near abolition, of NC1 binding—reflecting incompatibility—while also acknowledging that co-binding could, in principle, occur and produce an electrophysiological profile similar to $Ca^{2+}$ alone.

*Reviewer #3 (Remarks to the Author):*

*Revisiting the minimal established knowledge of TRPM4 pharmacology through the lens of physiological temperature has clearly been a thoughtful and enlightening endeavor. The authors have identified TPPO as a TRPM4 activator whose activity is restricted to physiologically relevant temperatures and calcium concentrations. Whereas the agonist NC1 loses its potency at high calcium. They also observe that, although both agonists and antagonists bind within the S1–S4 bundle, they occupy distinct sites (ie. S1-S4high for agonists and S1-S4low for antagonists). The authors use patch-clamp electrophysiology, single particle cryo-EM, and structure-function analysis for this study. The findings are original, rigorous, and well presented.*

*Overall, the data quality is good, and the conclusions are consistent with the data. Furthermore, the results presented are of interest to those in the TRP channel, broader ion channel, and potential protein biochemistry fields.*

*Overall, I question the strong emphasis placed on the broad applicability of these findings (i.e., that temperature and calcium influence TRPM4 pharmacology) to proteins in general. As written, the manuscript does not present evidence supporting this generalization. For example, were similar approaches applied to other ion channels, and if so, did they yield comparable insights? If additional examples exist, incorporating them into the introduction or discussion would strengthen this claim. Alternatively, if such data are not available, I suggest tempering the claim accordingly and adding a statement in the discussion noting that testing the broader applicability of these observations would be an important avenue for future work.*

**Response:** We appreciate the reviewer's point regarding the scope of our conclusions. Modulation of ligand binding by temperature (and potentially by $Ca^{2+}$) has historically received limited attention, and consequently relatively few explicit examples are available in the literature. We have therefore tempered the language in the manuscript to avoid overgeneralization.

Nevertheless, precedent exists: crystallographic studies of proteins such as PBP1B and several metal–protein systems have reported temperature-dependent effects on ligand binding, and more recently, a study of ionotropic glutamate receptors has shown that glutamate-dependent pore opening is strongly promoted at physiological temperatures, allowing activated states to be captured by cryo-EM that were not observed in samples prepared under non-physiological temperatures. We have added these examples and the corresponding citations to the Discussion. We now frame the broader applicability of our findings as a testable hypothesis and note that extending similar approaches to other ion channels and proteins will be an important direction for future work (lines 452–470).

*I leave the following suggestions and comments for the authors consideration.*

*The introduction and discussion emphasize the importance of physiological temperature and calcium concentrations for mechanistic protein studies but gives comparatively little attention to the biological role of TRPM4. This emphasis feels somewhat unbalanced, and I suggest reorganizing these paragraphs to more clearly highlight TRPM4's physiological relevance.*

**Response:** In response to this suggestion, we have expanded the Introduction to better emphasize the physiological relevance of TRPM4 in addition to its mechanistic features (lines 68–95).

*The authors should better explain what is meant by Brugada syndrome and inherited conduction disorders, particularly for readers who may not be familiar with these conditions. The authors should also clarify whether TRPM4 agonists or antagonists are likely to be relevant for treating these diseases.*

**Response:** We thank the reviewer for this helpful suggestion. We have expanded the Introduction to provide a description of Brugada syndrome as a genetic cardiac arrhythmia.

In addition, we now clarify the potential therapeutic relevance of both TRPM4 antagonists and agonists. Specifically, TRPM4 antagonists may be beneficial in cardiac diseases where excessive TRPM4 activity contributes to pathological depolarization and conduction defects, whereas TRPM4 agonists could be useful in certain cancers in which TRPM4 is upregulated,

where enhanced channel activity may promote cancer cell death through necrotic pathways. Please see lines 76–82.

*Neither the text nor the figures link ligand binding, whether by activators or inhibitors, to structural changes in the pore. In the absence of the underlying maps and models, this aspect of the story remains missing. I suggest that this be remedied by explicitly addressing pore conformational changes. This will strengthen the completeness of the mechanistic interpretation.*

**Response:** Our primary focus is on how temperature and intracellular $Ca^{2+}$ shape ligand binding to TRPM4, rather than on gating transitions per se. Nevertheless, we agree that explicitly stating the pore conformation helps complete the mechanistic picture. Accordingly, we have added a new section (lines 434-440) describing the pore conformation observed in our structures. We clarify that the pore remains in a closed state under the conditions examined and provide a plausible explanation for the observation.

*Using your mutants, is it possible to generate channels that respond to NBA/CBA but not TPPO or NC1, and vice versa? This would help address how linked the pockets are.*

**Response:** This is an insightful suggestion. Because the NBA/CBA and TPPO/NC1 binding sites are in close proximity within the S1–S4 bundle, many interacting residues overlap. Nevertheless, guided by the structures, we selected residues predicted to interact preferentially with one ligand class but not the other, and then tested these mutations functionally to assess the degree of linkage between the two pockets.

Specifically, N786 interacts selectively with the TPPO/NC1 site. The N786A mutation completely abolished the effect of NC1 while leaving CBA/NBA inhibition intact, supporting partial independence between the two binding sites. We have added the N786A data to Extended Data Fig. 2c and included a corresponding discussion in the text (lines 363–367).

Conversely, W864 and Q1061 interact exclusively with the CBA/NBA site. However, the W864A and Q1061A mutants exhibit little to no current under basal $Ca^{2+}$ conditions required to assess NC1 effects and are not activated by NC1 under these conditions (Fig. R1, see below), in contrast to WT (Fig. 2e). This behavior suggests that these mutations may disrupt the integrity of the S1–S4 domain and impair channel activation more generally, rather than selectively affecting NC1 binding. Because these data do not allow us to unambiguously distinguish between these possibilities and do not provide additional insight into the degree of linkage between the NBA/CBA and TPPO/NC1 sites, we have not included them in the manuscript but provide them here for completeness.

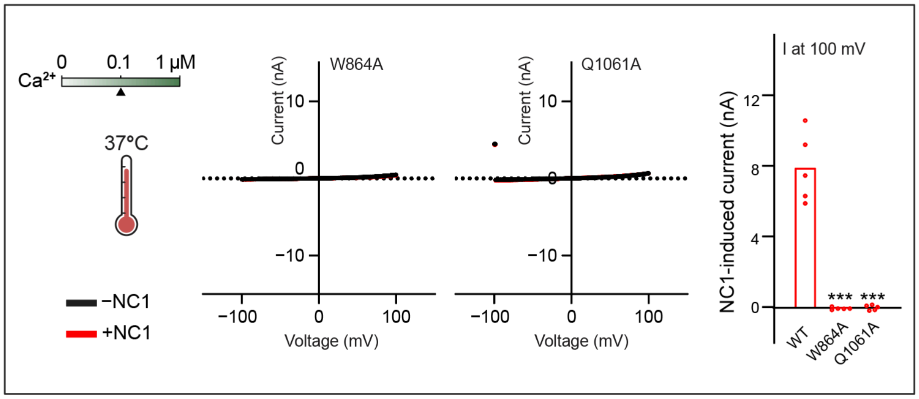

**Fig. R1.** Left: Recording conditions for the whole-cell electrophysiology experiments. Middle: Whole-cell currents recorded in the absence (black) and presence (red) of NC1 for the W864A and Q1061A mutants. Right: Comparison of NC1-induced currents measured at +100 mV in WT, W864A, and Q1061A mutants.

*The discussion of DVT positive modulation lacks sufficient introduction to explain what it is or why this experiment is informative.*

**Response:** We have added a brief explanation of the rationale for the DVT modulation experiment and clarified why it is informative for understanding the inhibition mechanism of NBA/CBA (lines 384–397).

*Line 365: Change the word protein to TRPM4. Is there another example of a protein whose ligand binding is modulated by temperature and/or calcium?*

**Response:** We have changed the word "protein" to "TRPM4". With respect to other examples, modulation of ligand binding by temperature (and potentially by $Ca^{2+}$) has historically received limited attention, and consequently relatively few explicit examples are available in the literature. Nevertheless, crystallographic studies of proteins such as PBP1b and several metal–protein systems have reported temperature-dependent effects on ligand binding. More recently, studies of AMPA receptors have shown that physiological temperatures are required for glutamate to induce pore opening at the structural level. We have added these examples and the corresponding citations to the Discussion (lines 459–470). We anticipate that our findings will motivate broader consideration of temperature (and $Ca^{2+}$) as key determinants of ligand engagement in ion channels and other proteins.

*Minor Comment:*

*Lines 180-181: Does this sentence have the intended meaning if you remove the word ‘where’?*

**Response:** We removed “where” as suggested.
